# Electroactive Carbazole-Based Polycyclic Aromatic Hydrocarbons: Synthesis, Photophysical Properties, and Computational Studies

**DOI:** 10.1021/acsomega.4c01434

**Published:** 2024-06-18

**Authors:** Hui Qi Wong, Ting-Hsuan Lin, Jian-Ming Liao, Septia Kholimatussadiah, Febri Baskoro, Hui-Hsu Gavin Tsai, Hung-Ju Yen

**Affiliations:** † Institute of Chemistry, Academia Sinica, 128 Academia Road, Section 2, Nankang, Taipei 11529, Taiwan; ‡ Sustainable Chemical Science and Technology Program, Taiwan International Graduate Program (TIGP), Academia Sinica and National Taiwan University, Taipei 11529, Taiwan; § Department of Chemical Engineering, National Taiwan University, Taipei 10617, Taiwan; ∥ Department of Chemical Engineering, National Taiwan University of Science and Technology, Taipei 10607, Taiwan; ⊥ Department of Chemistry, National Central University, No. 300, Zhongda Rd., Zhongli District, Taoyuan City 32001, Taiwan; # Nano Science and Technology Program, TIGP, Academia Sinica and National Taiwan University, Taipei 11529, Taiwan; ∇ Research Center of New Generation Light Driven Photovoltaic Module, National Central University, No. 300, Zhongda Road, Zhongli District, Taoyuan City 32001, Taiwan

## Abstract

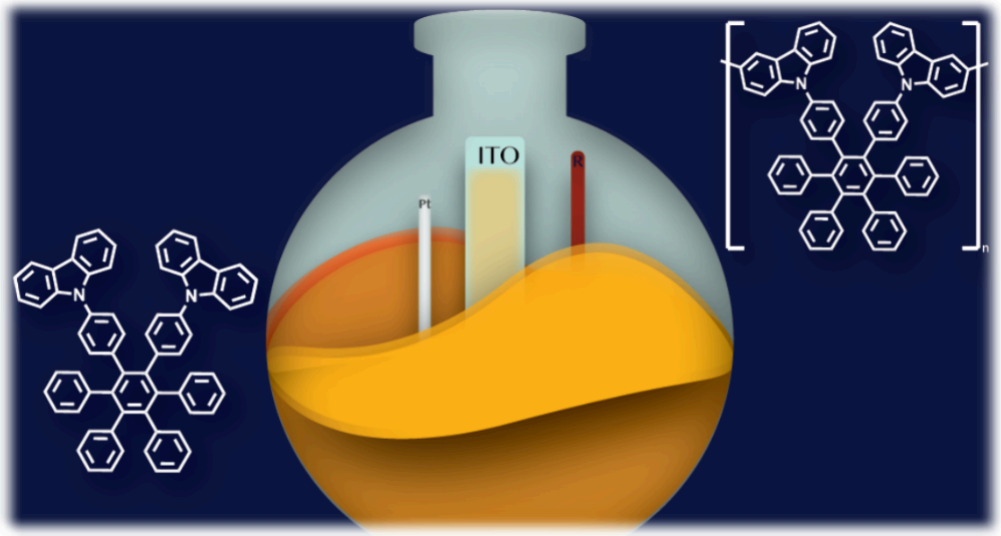

Herein, we explored the oxidative coupling reactions of carbazole-based polycyclic aromatic hydrocarbons using traditional Scholl reactions and electrochemical oxidation. Our findings indicate that the oxidation predominantly occurs at the carbazole functional group. The underlying reaction mechanisms were also clarified through theoretical investigations, highlighting that the primary oxidation pathway involves the 3,6-positions of the carbazole moiety, which is attributable to its high electron density.

## Introduction

Polycyclic aromatic hydrocarbons (PAHs) are neutral and nonpolar molecules. They were first discovered in the 19th century during the combustion of fossil fuels. They have garnered extensive attention in organic optoelectronics due to their intermolecular π–π stacking features, providing high charge carrier mobility, which is beneficial for various semiconductor applications. Furthermore, their remarkable properties of high specific surface area and high thermal and mechanical stabilities make them feasible for energy storage applications. Over the past decades, PAHs have expanded their categories and now integrated with other classes of materials, such as structurally extended graphene nanoribbons and covalent organic frameworks.^1,2^

The realization of polymerizing macromolecular sheets has been enabled by scientific contributions from many fields including supramolecular chemistry, framework chemistry, and crystal engineering. The 2D polymerization strategies have emerged as dominant approaches and can be divided into topochemical, on-surface solvent-free, and solution-based approaches, which can be further stimulated by their properties in specific applications in electrochemical energy storage and photo- and electrocatalysis. In addition to this, a great diversity of size, shape, or doping structurally well-defined PAH/frameworks can be synthesized through bottom-up organic synthesis or polymerizations.^3^ For example, it takes multiple steps to synthesize hexa-*peri*-hexabenzocoronene (HBC), the smallest fragment of a PAH precursor involving a strong acid and oxidant. The properties of PAHs can be adjusted through the molecular size, shape, and peripheral substituents, even though PAHs consist of all identical sp^2^-hybridized carbon atoms. A wide range of chemical and optical properties can be found depending on the edge-functionalized moieties on particular PAHs. Thus, PAHs have been explored as a popular topic in biology and organic chemistry and applied for optoelectrical devices for decades.

Throughout the overview of past published literature, the only drawback found from organic synthesis is that oxidation reactions might fail unpredictably for some high electron density structures.^4^ Apart from classical organic synthesis of PAHs, the electro-organic synthesis method was commonly employed for C–C bond formation, rendering chemical oxidizing agents unnecessary.^5^ Moreover, it significantly addresses solubility issues during experimentation, and the time scale can be reduced compared with conventional methods. Ma et al. demonstrated electrochemical cyclodehydrogenation and electrochemical deposition of hexaphenylbenzene (HPB) to HBC on indium tin oxide (ITO). Consequently, the electro-organic synthesis method has emerged as an alternative approach for bottom-up synthesis of HBC. The electro-organic synthesis method not only avoids the use of strong acids and oxidants but also prevents the intermolecular rearrangement of HBC.^4−7^

In fact, the application of HBCs is still limited due to the finite optical band gap inherent in their structure. Thus, the physicochemical, optical, electromagnetic, and structural properties of HBCs can be modulated by inducing heteroatoms, a topic that has been widely studied and implemented in various applications.^8^ The number and kind of heteroatoms can depend on the requirements of the application, such as nitrogen, boron, phosphorus, or sulfur, among others. All of these HBCs play roles as excellent electrochemical materials. Carbazole and its derivatives have been studied over the years, showing great potential as polymers and materials for optoelectronics due to their redox-active properties.^9−12^ With both the π-conjugated system and the presence of a nitrogen atom on the carbazole moiety, it aids in increasing the rigidity of the structure and exhibits high electron mobility capabilities.^13−15^ Based on the aforementioned reasons, incorporating the carbazole functional group into HBCs might improve the structural stability of the material while enhancing electron mobility, resulting in an appropriate HOMO–LUMO energy band gap suitable for optoelectronic applications.

Oxidative polymerization of carbazole can be classified into two methods: Scholl oxidation reaction^16,17^ or through electropolymerization on ITO glass as an electrode.^18,19^

Consequently, the simultaneous reaction of oxidative C–C coupling of HPB and electropolymerization of the carbazole moiety, facilitated by electrochemistry, will effectively reduce the number of synthetic steps. In this work, studies on oxidation with various Scholl oxidative conditions and electrochemical cyclodehydrogenation have been conducted on carbazole-based HPB. All of the products were characterized, and the reaction mechanisms were supported by simulation.

## Results and Discussion

### Synthesis and Characterization

**Scheme 1:**
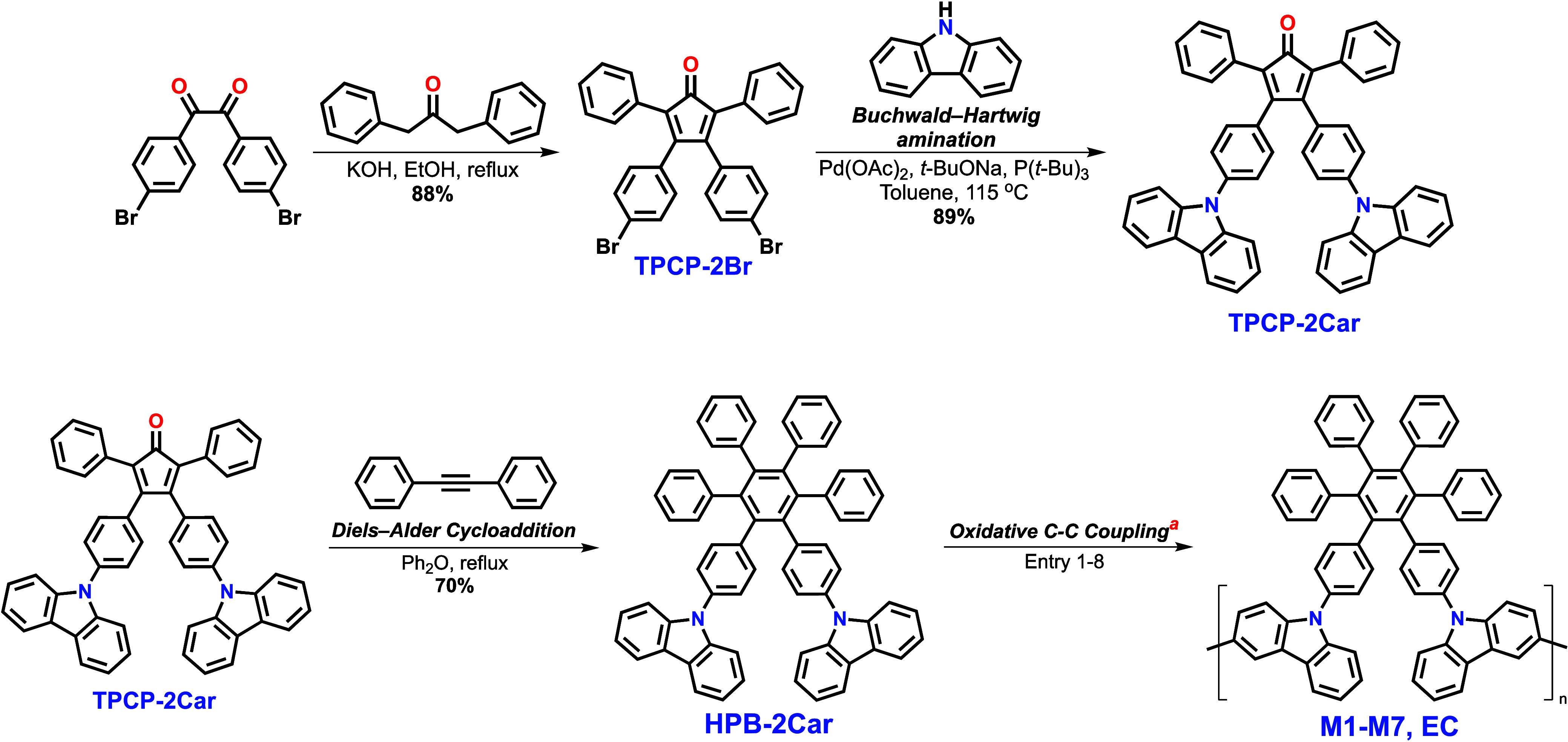
Synthetic Route to **HPB-2Car** and Further Oxidative Coupling Reactions Condition of entry: (1) MoCl_5_, CH_2_Cl_2_, r.t.; (2) AlCl_3_, CuCl_2_, CS_2_, r.t.; (3) FeCl_3_, CH_2_Cl_2_, CH_3_NO_2_, r.t.; (4) FeCl_3_, 1,2-dichoroethane, CH_3_NO_2_, 80 °C; (5) AlCl_3_, Cu(SO_3_CF)_2_, CS_2_, r.t.; (6) DDQ, triflic acid, CH_2_Cl_2_, r.t.; (7) PhI(O_2_CCF_3_)_2_, BF_3_·Et_2_O, CH_2_Cl_2_, r.t.; (8) electrolyte: 0.1 M TBAP, CH_2_Cl_2_.

**HPB-2Car** was synthesized with a high overall yield as high as 55%, as illustrated in Scheme 1. Initially, **TPCP-2Br** was synthesized via aldol condensation of 4,4′-dibromobenzil and 1,3-diphenylacetone under basic conditions followed by Buchwald–Hartwig amination with 9*H*-carbazole to obtain **TPCP-2Car**. The subsequent Diels–Alder reaction of **TPCP-2Car** and diphenylacetylene was used to obtain **HPB-2Car**. All of the intermediates were purified and characterized by NMR and mass spectroscopic techniques (Figures S1–S6).

Oxidative cyclodehydrogenation reactions are commonly exploited for the final step in the synthesis of PAHs such as HBC. The pioneering work of this synthetic chemistry was already reported by Scholl et al. over 100 years ago.^20^ Oxidative cyclodehydrogenations commonly proceed in the presence of Lewis acids, such as AlCl_3_ or FeCl_3_, and are nowadays frequently called Scholl reactions.^21,22^

**Figure 1:**
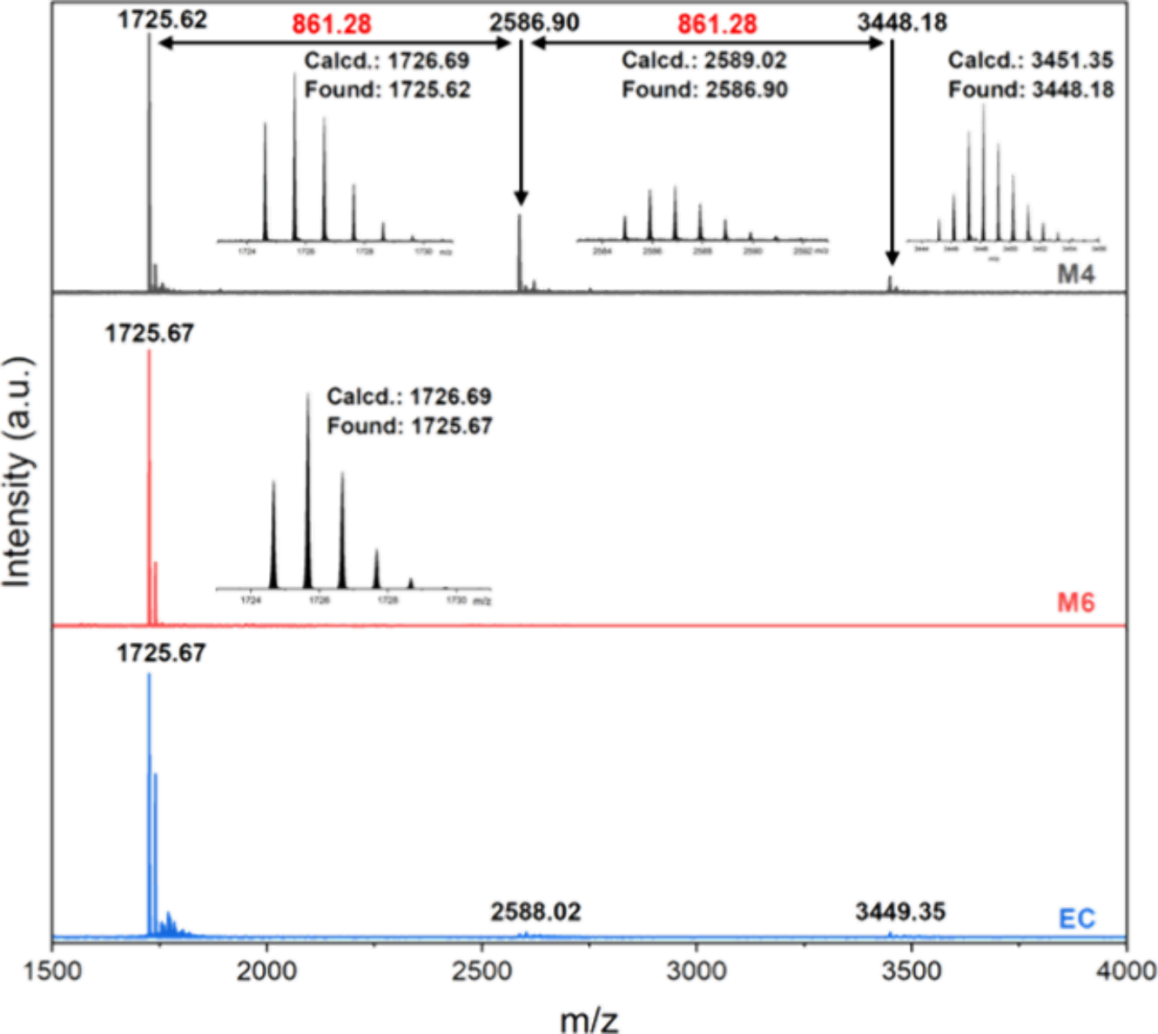
MALDI-TOF MS spectra of **M4**, **M6**, and **EC**.

To get one step closer to the unprecedented carbazole-based PAHs, typical Scholl reaction conditions were applied to **HPB-2Car**. All reaction outcomes obtained for the conversion of **HPB-2Car** toward carbazole-based HBC were investigated via MALDI-TOF MS (Figure 1, Figures S7–S14) and are summarized in Table 1. Initially, cyclodehydrogenation reactions with the MoCl_5_ and AlCl_3_/CuCl_2_ system were performed at room temperature as **M1** and **M2**, respectively. It was revealed via MALDI-TOF MS that both reaction conditions did not yield the desired target.

**Table 1:** Summary of the Oxidative Coupling Approaches

sample	entry	reaction condition	outcome
**M1**	1	MoCl_5_, CH_2_Cl_2_, r.t., O/N	no target found on mass
**M2**	2	AlCl_3_, CuCl_2_, CS_2_, r.t., 5 days	no target found on mass
**M3**	3	FeCl_3_, CH_2_Cl_2_, CH_3_NO_2_, r.t., 13 days	*m*/*z* 1725.6, 2586.9, 3448.2
**M4**	4	FeCl_3_, 1,2-dichoroethane, CH_3_NO_2_, 80 °C, 7 days	*m*/*z* 1725.6, 2586.9, 3448.2
**M5**	5	AlCl_3_, Cu(SO_3_CF)_2_, CS_2_, r.t., 13 days	*m*/*z* 1725.6, 2586.9, 3448.2
**M6**	6	DDQ, triflic acid, CH_2_Cl_2_, r.t., 12 days	*m*/*z* 1725.6
**M7**	7	PhI(O_2_CCF_3_)_2_, BF_3_·Et_2_O, CH_2_Cl_2_, −40 °C to r.t., 3 h	*m*/*z* 1725.6 found but with impurity
**EC**	8	electrolyte: 0.1 M TBAP in CH_2_Cl_2_	*m*/*z* 1725.6

Thereby, different reaction conditions and reagent systems were chosen for further oxidative attempts (**M3** to **M7**). The complete consumption of the starting material was indicated in all entries via TLC and subsequent MALDI-TOF MS analyses of the crude reaction mixtures. The reaction was quenched with methanol, and the precipitate was filtered off and repeatedly washed with methanol. Due to the insolubility of synthesized oligomers, no attempt is made to isolate or purify the products. After the workup of the reaction mixtures, MALDI-TOF MS depicted the presence of a major product associated with a mass of *m*/*z* 1725.6, indicating dimerization occurred throughout **M3** to **M7**. Additionally, the presence of ion peaks at *m*/*z* = 2586.9 and 3448.2 for **M3**–**M5** indicated the formation of the trimer and tetramer in these three oxidative coupling conditions. A signal of the dimer was also observed in the MS spectra at *m*/*z* 1725.6 for **M7** but with impurities.^23^ Unfortunately, all attempts did not result in the formation of the desired carbazole-based HBC, even though the reaction temperatures and times were carefully adjusted. Finally, electro-organic synthesis was also applied to **HPB-2Car** using cyclic voltammetry (entry 8, termed as **EC**), which has been reported as an efficient method to proceed with cyclodehydrogenation and electrochemical deposition of HPB to HBC.^6^

**Figure 2:**
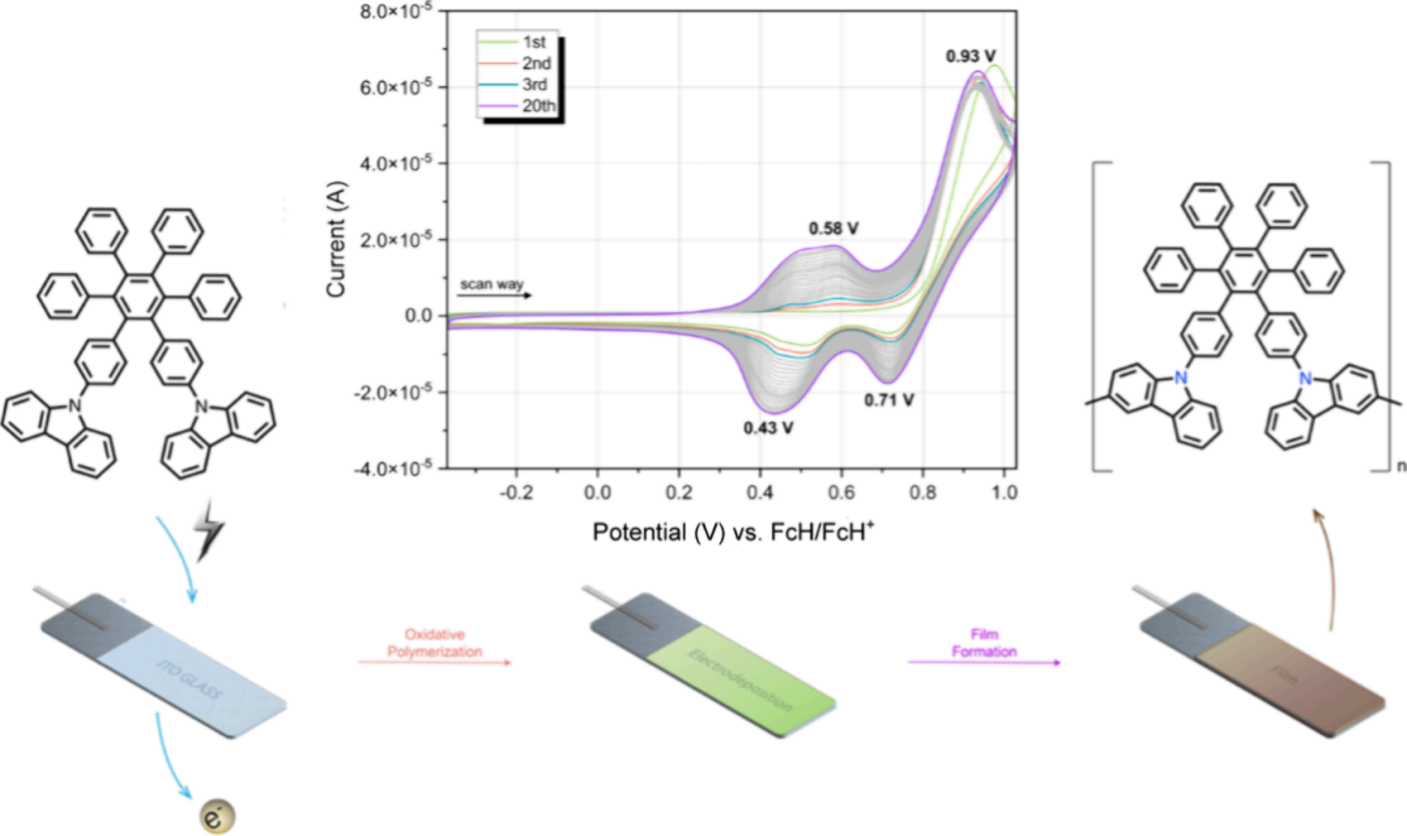
CV curves of **HPB-2Car** on ITO were recorded in 0.1 M TBAP/CH_2_Cl_2_ at 50 mV/s.

The electrochemical oxidation of **HPB-2Car** was conducted by using cyclic voltammetry (1.0 mM in 0.1 M TBAP/CH_2_Cl_2_ electrolyte solution) in a three-electrode system, with ITO-coated glass serving as the working electrode, a platinum wire as the counter electrode, and a Ag/AgCl reference electrode. Figure 2 displays the CV scans and a schematic representation of the electro-oxidative process for a solution containing 1.0 mM **HPB-2Car** on an ITO electrode, cycled repetitively from 0 to 1.05 V vs FcH/FcH^+^ (FcH: ferrocene) for 20 cycles at a scan rate of 50 mV/s. For the first positive potential scan, we observe one oxidation wave (anodic peak) at about 0.95 V, with two reduction waves in the cathodic scan (cathodic peaks at around 0.43 and 0.71 V). It is known that the carbazole moiety can be oxidized to the respective monoradical cation.^24^ In the second scan, a new oxidation peak appeared at a lower potential of 0.45–0.6 V, which might be associated with the oxidation processes of the biscarbazole structure. Furthermore, upon repetitive scanning of the solution of **HPB-2Car** over the voltage range from 0 to 1.05 V, new redox patterns were found to grow in intensity on the electrode. Additionally, with an increase in CV scanning cycles, two new redox waves at 0.45–0.6 V and 0.74–0.95 V emerged, and their current intensity gradually increased. This suggested the deposition of oligomers of **HPB-2Car** on the ITO electrode. During the electrochemical oxidation process, the color of the solution surrounding the ITO working electrode turned light brown with oxidation and returned to the original solution color with reduction, indicating reversible redox properties. Regarding electrochemical performance, we also observed evident electropolymerization characteristics of **HPB-2Car**. Therefore, we utilized the amperometry mode to maintain a stable voltage during the electrochemical synthesis process, ensuring that the film was indeed deposited on the ITO working electrode. Ultimately, we identified this electrodeposited film as the dimer of **HPB-2Car** through MALDI-TOF MS using a simple electrochemical synthesis method (Figure S14).

### Theoretical Investigations of Scholl Reaction Mechanisms of **HPB-2Car**

**Scheme 2:**
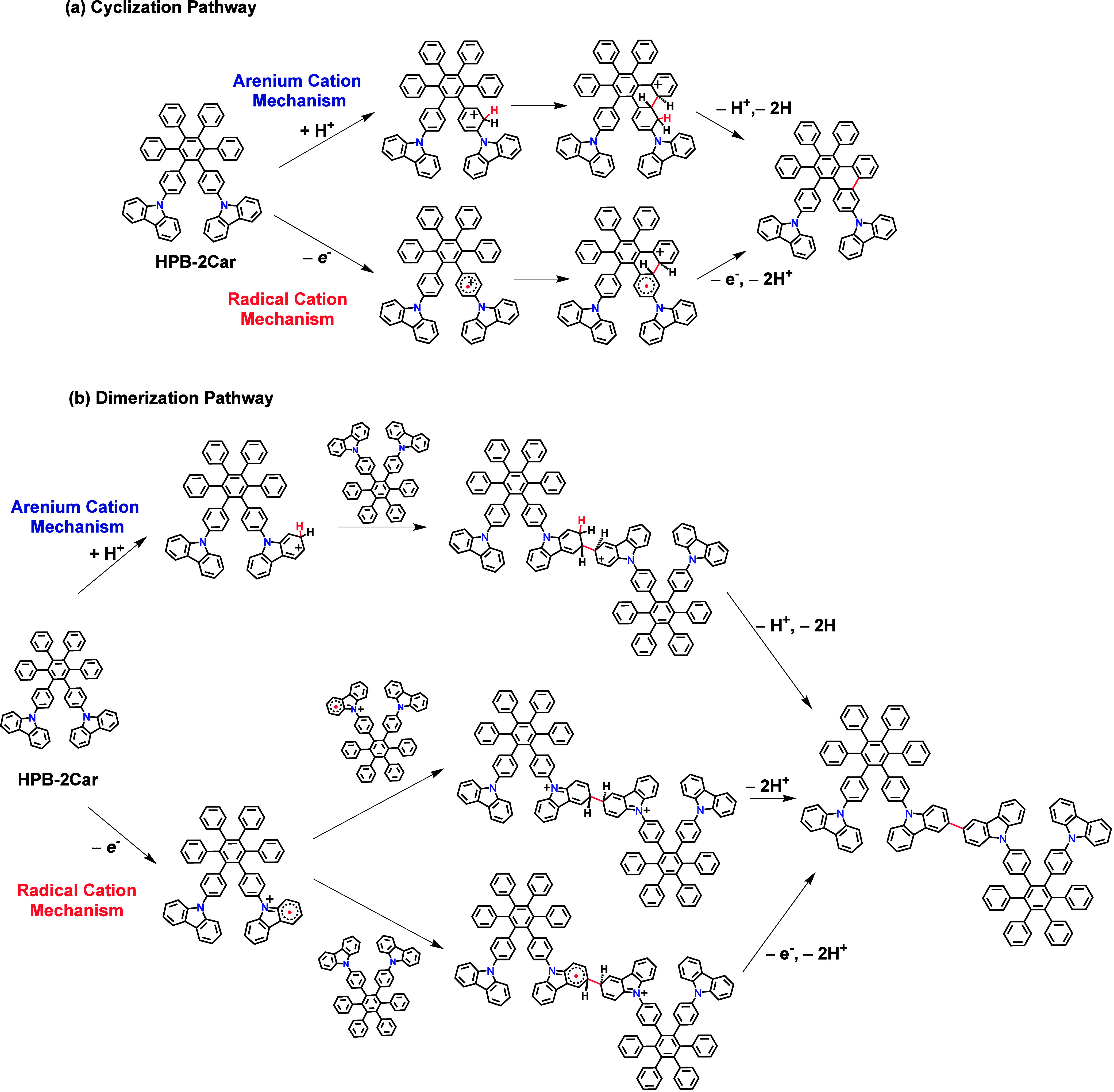
Proposed Reaction Pathways for (a) Cyclization and (b) Dimerization of **HPB-2Car** Involving the Arenium Cation and Radical Cation Mechanisms

To better understand the Scholl reaction mechanisms^25^ of **HPB-2Car**, this study initially proposes two distinct reaction pathways: cyclization and dimerization (refer to Scheme 2). Moreover, these two pathways can manifest themselves via two distinct mechanisms, namely, the arenium cation mechanism and the radical cation mechanism.^26−28^ Taking DDQ and triflic acid as reaction agents, in the case of the arenium cation mechanism, the initial protonation event occurs with triflic acid (CF_3_SO_3_H) serving as the proton donor, subsequently followed by dehydrogenation facilitated by DDQ as the acceptor for the two hydrogen atoms. On the other hand, in the radical cation mechanism, DDQH^+^ acts as the electron acceptor, leading to the participation of the DDQH**·** radical as the acceptor for hydrogen (H), while CF_3_SO_3_^–^ functions as the acceptor for H^+^.

#### Computational Methods

To investigate their energy profiles, density functional theory (DFT) calculations were conducted. All calculations were performed using Gaussian 16^29^ at the B3LYP/6-31G(d,p) level, with the inclusion of the D3 dispersion correction^30^ for the closed-shell systems, while the unrestricted UB3LYP/6-31G(d,p) level was applied for the open-shell radical systems. The C-PCM model^31^ was employed to consider the solvation effects in dichloromethane. All stationary states were confirmed through vibrational frequency calculations, ensuring the presence of only positive vibrational frequencies. Likewise, transition states were verified by vibrational frequency calculations, displaying a single negative vibrational frequency, and further validated by examining their intrinsic reaction coordinate to determine the corresponding reactants and products.

#### Reaction Sites of Protonation

**Figure 3:**
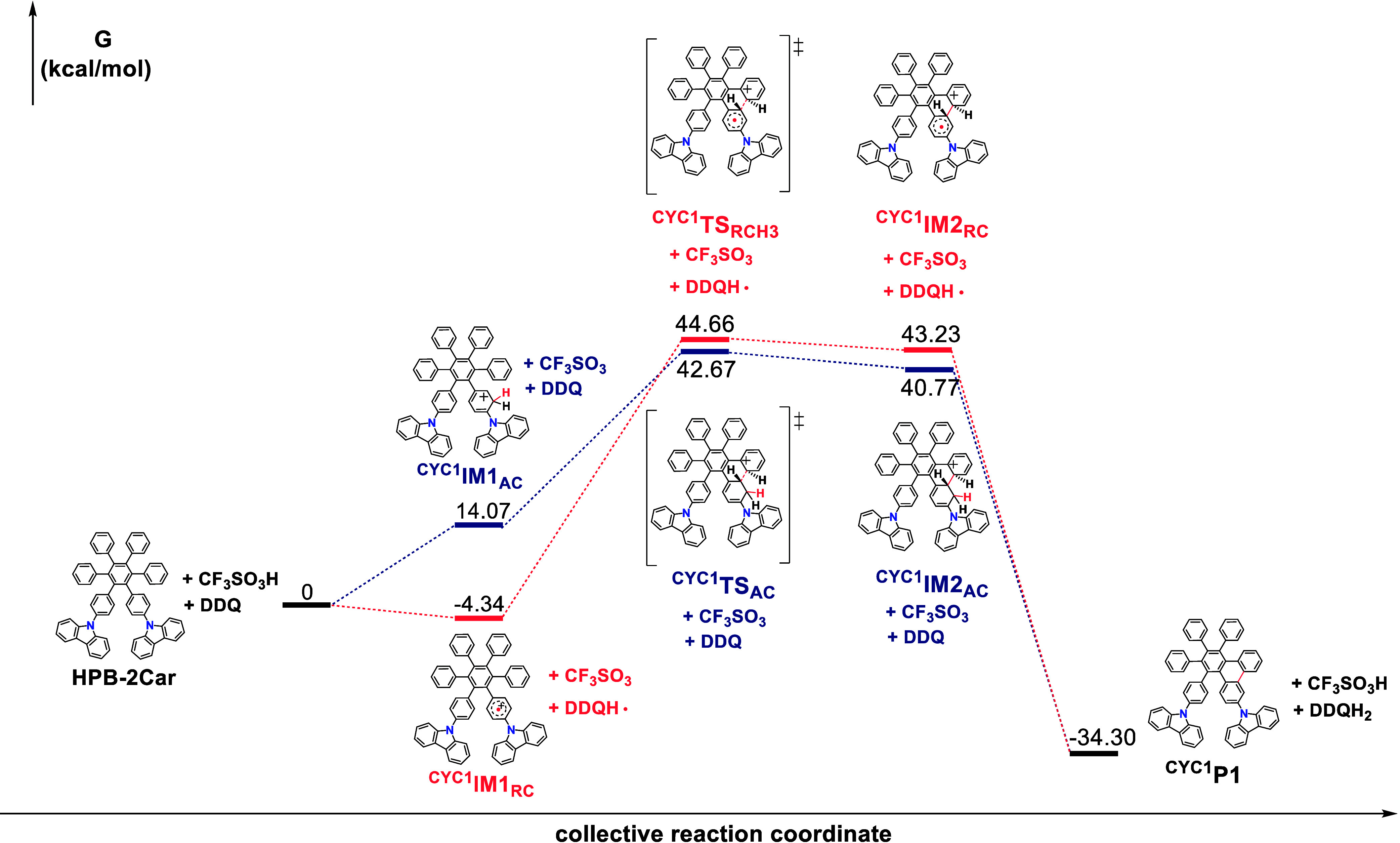
DFT calculated relative Gibbs free energies. (Δ*G,* at 298 K) for the cyclization pathway 1 of **HPB-2Car** involving the arenium cation (blue bars) and radical cation (red bars) mechanisms.

**Figure 4:**
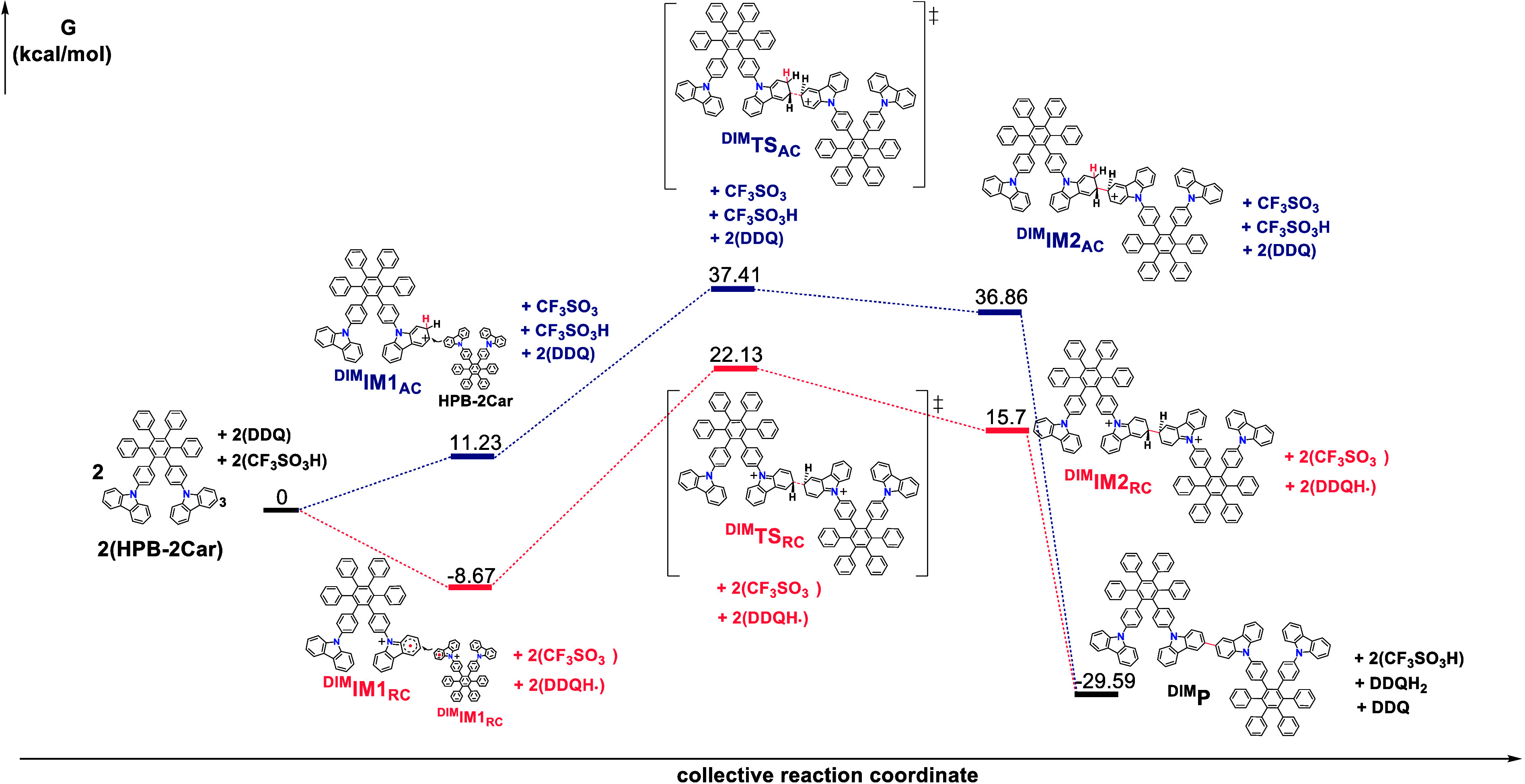
DFT calculated relative Gibbs free-energies. (Δ*G*, at 298 K) for the dimerization pathway of **HPB-2Car** involving the arenium cation (blue bars) and radical cation (red bars) mechanisms.

In the context of the arenium cation mechanism, CF_3_SO_3_H has the ability to protonate various carbons of **HPB-2Car**. To investigate the relative stabilities of the different isomers of protonated **HPB-2Car**, we initially assessed eight different isomers of protonated **HPB-2Car** (refer to Figure S15). Our calculations reveal that protonation at position 3 of the carbazole moiety corresponds to a relative energy of 0.0 kcal/mol, while protonation at position 4 has a relative energy of 1.08 kcal/mol. Additionally, the subsequent two most stable isomers possess relative energies of 2.84 and 5.63 kcal/mol, respectively, resulting from protonation on the phenyl group adjacent to the carbazole. Conversely, the remaining four protonated **HPB-2Car** isomers exhibit higher energies (>8 kcal/mol), rendering them thermodynamically inaccessible. Regarding the oxidative coupling of carbazoles via the Scholl reaction, previous studies have demonstrated that intermolecular oxidative coupling of 9-arylcarbazoles can occur, while intramolecular reactions may exclusively and quantitatively produce 3,3′-bicarbazyl.^32^ This is consistent with our DFT calculations, which suggest that protonation at position 3 of the carbazole moiety results in the lowest energy isomer. Consequently, we select the most stable isomer (see Figure 4, ^**DIM**^**IM1**_**AC**_) for subsequent dimerization calculations, while the third most stable isomer (see Figure 3, ^**CYC**^**IM1**_**AC**_) is chosen for the subsequent cyclization calculations.

#### Reaction Mechanism of Cyclization

Figure 3 illustrates the computed energy profiles (Gibbs free energy) for the cyclization of **HPB-2Car** through the arenium and radical cation mechanisms. In the arenium cation mechanism, CF_3_SO_3_H protonates **HPB-2Car**, leading to the formation of intermediate ^**CYC**^**IM1**_**AC**_, which is 14.07 kcal/mol higher in Gibbs free energy compared with the reactants. Subsequently, ^**CYC**^**IM1**_**AC**_ can undergo an electrophilic reaction via transition state ^**CYC1**^**TS**_**AC**_ (cyclization pathway 1), resulting in the formation of cyclized intermediate ^**CYC1**^**IM2**_**AC**_, which is slightly more stable (1.90 kcal/mol) than ^**CYC1**^**TS**_**AC**_. Following deprotonation and oxidative dehydrogenation, the cyclized product ^**CYC1**^**P** is produced, exhibiting stability that is 34.30 kcal/mol higher than that of the reactants. The overall activation energy for the cyclization pathway 1 via the arenium cation mechanism is calculated to be 42.67 kcal/mol. Moreover, ^**CYC**^**IM1**_**AC**_ can also undergo an electrophilic reaction via the transition state ^**CYC2**^**TS**_**AC**_ (cyclization pathway 2, refers to Figure S17), resulting in the formation of the cyclized intermediate ^**CYC2**^**IM2**_**AC**_, which is slightly more stable (0.51 kcal/mol) than ^**CYC2**^**TS**_**AC**_. The overall activation energy for the cyclization pathway 2 via the arenium cation mechanism is calculated to be 43.56 kcal/mol. Cyclization pathway 1 has a slightly lower activation energy than cyclization pathway 2.

On the other hand, in the radical cation mechanism, DDQH^+^ serves as the electron acceptor. DDQH^+^ is generated by protonating DDQ with CF_3_SO_3_H. DDQH^+^ oxidizes **HPB-2Car**, leading to the formation of radical intermediate ^**CYC**^**IM1**_**RC**_, which is −4.34 kcal/mol more stable than the reactants. Subsequently, cyclization of ^**CYC**^**IM1**_**RC**_ occurs through transition state ^**CYC1**^**TS**_**RC**_ (cyclization pathway 1), resulting in the formation of the cyclized intermediate ^**CYC1**^**IM2**_**RC**_. Following one-electron oxidation and 2-fold deprotonation processes, the cyclized product ^**CYC1**^**P** is obtained. The overall activation energy for cyclization pathway 1 via the radical cation mechanism is calculated to be 44.66 kcal/mol, which is comparable to that of the corresponding arenium cation mechanism (42.67 kcal/mol). On the other hand, cyclization of ^**CYC**^**IM1**_**RC**_ can also occur through the transition state ^**CYC2**^**TS**_**RC**_ (cyclization pathway 2, refer to Figure S17), resulting in the formation of the cyclized intermediate ^**CYC2**^**IM2**_**RC**_. The overall activation energy for cyclization pathway 2 via the radical cation mechanism is calculated to be 45.76 kcal/mol.

#### Reaction Mechanism of Dimerization

Figure 4 depicts the computed energy profiles (Gibbs free energy) for the dimerization of **HPB-2Car** through the arenium and radical cation mechanisms. In the context of the arenium cation mechanism, we investigate the protonation at the 3-position carbon of the carbazole groups, resulting in the formation of the intermediate ^**DIM**^**IM1**_**AC**_, which is 2.84 kcal/mol more stable than its counterpart, ^**CYC**^**IM1**_**AC**_. These findings indicate that the carbazole groups are more prone to protonation compared to the phenyl group at the HPB. The dimerization between ^**DIM**^**IM1**_**AC**_ and **HPB-2Car** occurs via transition state ^**DIM**^**TS**_**AC**_, leading to the formation of intermediate ^**DIM**^**IM2**_**AC**_. The overall activation energy for the dimerization through the arenium cation mechanism is calculated to be 37.41 kcal/mol, which is 5.26 kcal/mol lower than the corresponding cyclization pathway 1. Furthermore, ^**DIM**^**IM2**_**AC**_ is 0.55 kcal/mol more stable than ^**DIM**^**TS**_**AC**_. In terms of carbazole oxidative coupling, previous studies have proposed two mechanisms: one involving a single radical cation and the other involving two radical cations.^33^ In this study, we examine both of these mechanisms. Our DFT calculations failed to identify an intermediate for dimerization of one ^**DIM**^**IM1**_**RC**_ and one **HPB-2Car** (refer to Figure S16). Instead, we observe the presence of an intermediate (^**DIM**^**IM2**_**RC**_) involving two ^**DIM**^**IM1**_**RC**_ radical cations as the stationary state. ^**DIM**^**IM2**_**RC**_ is 15.7 kcal/mol higher than those of the reactants. The overall activation energy for dimerization via the radical cation mechanism, specifically involving two ^**DIM**^**IM1**_**RC**_ radical cations, is determined to be 22.13 kcal/mol.

Taken together, our DFT calculations indicate that the dimerization of **HPB-2Car** exhibits a higher kinetic rate compared to cyclization. The overall activation energy for dimerization is lower than that for cyclization, regardless of whether it occurs through the arenium cation or radical cation mechanism. In the radical cation mechanism, the overall activation energy for dimerization involving two radical cations is 22.53 kcal/mol lower in Gibbs free energy than the corresponding initial cyclization reaction (cyclization pathway 1). These findings collectively suggest that dimerization of **HPB-2Car** is kinetically favorable over cyclization, which supports the experimental results.

**Figure 5:**
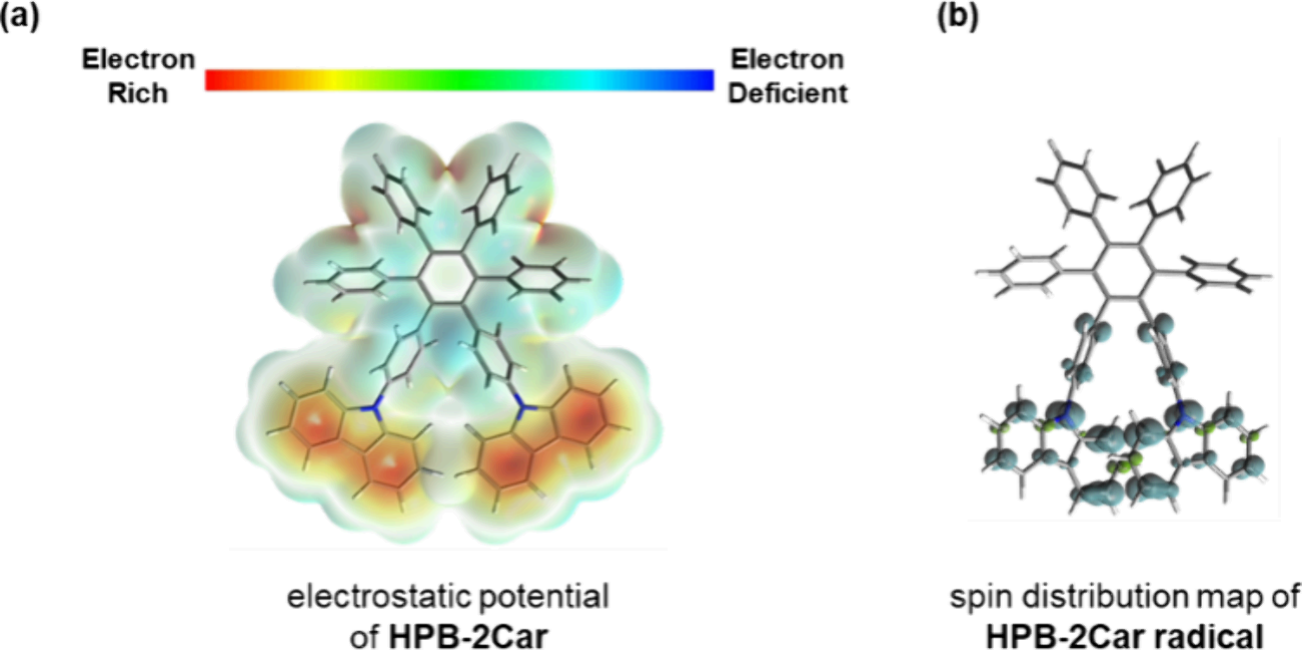
(a) Electrostatic potential and Mulliken charge densities of **HPB-2Car** (red: carbazole group; blue: phenyl group). (b) Calculated spin distribution map and spin density of **HPB-2Car** radical cation (red: carbazole group; blue: phenyl group).

To gain insights into the preferential reactivity of carbazole over the phenyl group in the Scholl reaction, we conducted calculations on the electrostatic potential (ESP; Figure 5a) of the stationary state of **HPB-2Car** and the spin density distribution (Figure 5b) of the stationary state of the **HPB-2Car** radical cation. The ESP depicted in Figure 5a reveals that the carbazole group of **HPB-2Car** exhibits a higher electron density compared with the phenyl groups. This indicates that the initial protonation in the arenium cation mechanism is more favorable to occur on the carbazole group. Additionally, Figure 5b shows that the spin density of the **HPB-2Car** radical cation is predominantly localized in the carbazole groups rather than the phenyl groups. This suggests that the radical cation mechanism preferably involves the carbazoles.

With the support of theoretical calculation results, as the electron distribution of **HPB-2Car** is mainly concentrated on the carbazole substituent, this highly active position takes priority for oxidation during the oxidation reaction. The results are fully consistent with our experimental outcomes.

### Basic Characterizations and Morphological Analysis

The FTIR data of **M3**-**M7** and **EC** are shown in Figure S18, and those of **TPCP2-Car**, **TPCP-2Br**, and **HPB-2Car** are shown in Figure S19. From this data, a typical IR spectrum of **HPB-2Car** and representative **M4**, **M6**, and **EC** exhibited characteristic absorption bands at around 1515 cm^–1^ (C=C stretch), 1450 cm^–1^ (C–H bending), and 1225 cm^–1^ (C–N stretching). Meanwhile, the dimerized products **M4**, **M6**, and **EC** do not contain a peak at 3050 cm^–1^, which could be attributed to the linkage between two **HPB-2Car** molecules, possibly through their aromatic sites 3- and/or 6-positions of carbazoles obtained both chemically and electrochemically.

Figure S20 shows the Raman spectra of **TPCP-2Br**, **TPCP-2Car**, **HPB-2Car**, **M3**, and **M6**. As shown in Figure S20, the specific vibrational mode of C=O from **TPCP-2Br** is monitored at 1710 cm^–1^, while the C=C mode (G band) can be monitored at 1605 cm^–1^. Additional vibration mode of C–N–C is observed at 640 cm^–1^, when the carbazole functional group is attached on TPCP, suggesting a scuccesful formation of **TPCP-2Car**.^34^ Interestingly, this vibrational mode of C–N–C was slightly shifted from 640 cm^–1^ to around 710 cm^–1^ after the Diels–Alder cycloaddition reaction to form **HPB-2Car** (Figure S20; blue line), which is possibly due to the larger π-conjugated system. Furthermore, the vibrational mode of C=O at 1710 cm^–1^ had disappeared after the cycloaddition reaction, further suggesting successful formation of the **HPB-2Car** monomer. Notably, the Raman spectrum was significantly changed compared to that of **HPB-2Car** after forming a dimer structure, such as **M6**, showing two prominent peaks associated with the D band (1345 cm^–1^) and G band (1605 cm^–1^) (Figure S20; green line). Moreover, the vibrational mode of C–N–C still can be monitored as a broad peak at ∼730 cm^–1^. Additionally, the vibrational mode of C–N–C was further minimized when it formed a mixed oligomer of dimer, trimer, and tetramer, such as **M3**, depicting only vibration mode of D and G bands (Figure S20; purple line). This phenomenon could be associated with the formation of a large π-conjugated system in the bulk structure consisting of different oligomers, possibly costacking, thus decreasing the intensity of the vibrational mode of the C–N–C bond. In brief, the Raman spectroscopy further confirmed the successful synthesis process of a series of carbazole-based oligomers, which is consistent with the FTIR data.

The SEM micrographs in Figure S21 reveal the differences between chemical and electrochemical dimerization and their monomer **HPB-2Car**. The morphology of all the samples was studied with the same magnification. Compared with the clear and well-packed crystal-like structure of **HPB-2Car**, **M4** and **M6** showed some clusters of globules, which are much different with the sponge-like (or coral-like) **EC** structure. The presence of microporosity in **EC** is expected to facilitate the counterion diffusion between electroactive species and electrolytes for further electrochemistry-related applications.

### Thermal and Optical Properties

The thermal stability of representative **M4**, **M6**, and **EC** was examined by using thermogravimetric analysis (TGA). TGA measurements were carried out by monitoring the changes in weight before and after heating samples with approximately 5 mg of flowing nitrogen (flow rate = 20 cm^3^/min) at a heating rate of 10 °C/min. Figure S22 shows the TGA curves of the samples, with decomposition temperatures (Td;^5^ 5% weight loss) recorded at 352 °C for **M4**, 405 °C for **M6**, and 595 °C for **EC** in nitrogen. The amount of carbonized residue (char yield) of the materials in a nitrogen atmosphere was more than 60% at 800 °C, with limiting oxygen index values up to 50. These samples exhibit excellent thermal stability up to 300 °C under a nitrogen atmosphere without significant mass loss. The high char yields of these samples could be attributed to the high aromatic content built into the structures.

**Figure 6:**
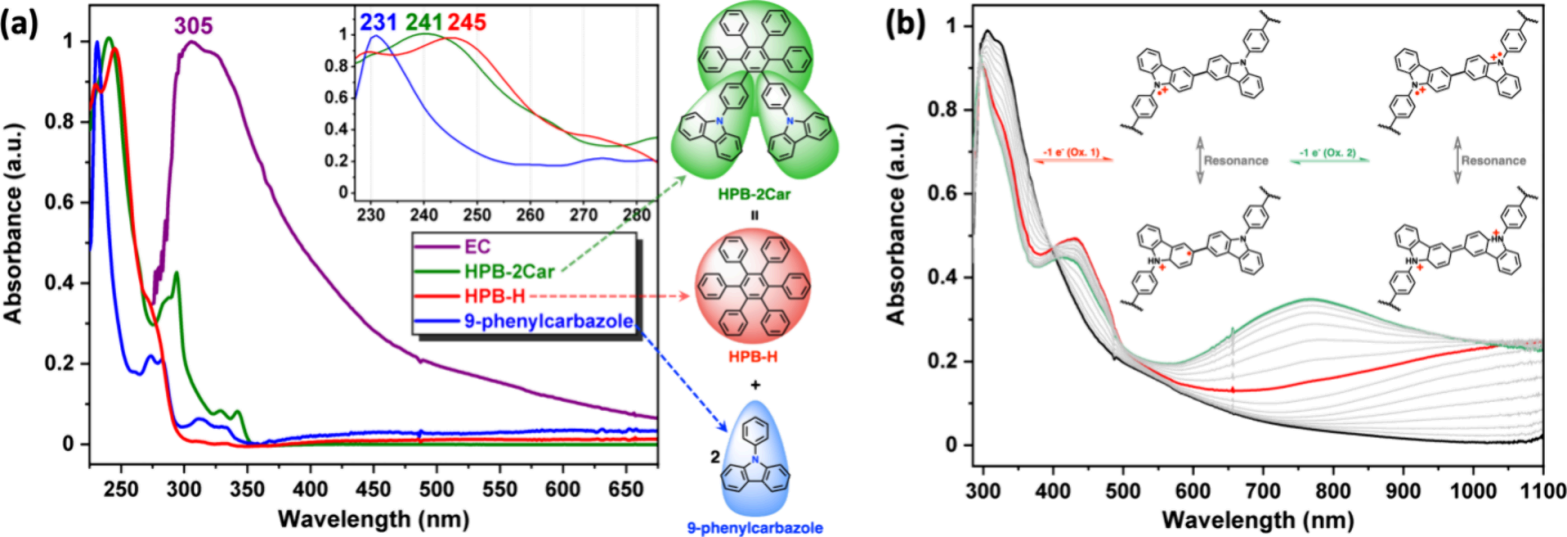
(a) Normalized absorbance spectra of **HPB-2Car**, HPB-H, and 9-phenylcarbazole in CH_2_Cl_2_ (10 μM) with the **EC** film. (b) Spectroelectrochemistry of the electrodeposited **EC** film by increasing the applied voltage to 1.0 (V vs Ag/AgCl) and its oxidation pathway.

The photophysical properties of the electrodeposited **EC** film have been studied by using UV–vis spectroscopy, and a comparison was made with hexaphenylbenzene (HPB-H), 9-phenylcarbazole, and **HPB-2Car**. The UV–vis spectra of the polycyclic aromatics were recorded in dichloromethane (concentration: 10^–5^ mol/L). As shown in Figure 6a, the UV–vis absorption of **HPB-2Car** was entirely derived from the superposition of reference materials HPB-H and 9-phenylcarbazole, revealing that there is little to no interaction between HPB and carbazole moieties within **HPB-2Car**. Nevertheless, despite the sizable torsions, the shifted absorbance onset toward a lower energy appreciably indicates the conjugation plane expansion from 9-Ph (9-phenylcarbazole) to HPB (**HPB-2Car**).

### Electrochemical and Electrochromic Properties

The work functions of the prepared materials were measured and are summarized in Figure S23. Compared with **HPB-2Car**, dimerized products possess a lower work function (around 5.50 eV), demonstrating a lower energy barrier for releasing electrons due to their conjugated skeleton. Furthermore, the reversible oxidation redox wave of **EC**, as shown in Figure 2, represents the formation of a stable radical cation originating from the electrochemical redox reactions of 3,3′-bicarbazole. During the electrochemical oxidation of the thin films, the color of the film changed from colorless to green. Therefore, spectroelectrochemical experiments were used to evaluate the optical properties of the deposited **EC** films. UV–vis absorbance curves correlated to applied potentials of films are depicted in Figure 6b. The strong absorption of **EC** at around 305 nm is characteristic of the triarylamine unit in neutral form (0 V). Upon oxidation (increasing applied voltage from 0 to 0.90 V), the intensity of the absorption peak at 305 nm gradually decreased, while a new peak at 430 nm and a broad IV-CT band centered around 1050 nm in the NIR region gradually increased in intensity. We attribute the spectral change in the visible light region to the formation of a stable monocation radical of the carbazole center in the 3,3′-bicarbazole moiety. Furthermore, the broad absorption in the NIR region is characteristic for IV-CT excitation^35−37^ between states in which the positive charge is centered at different nitrogen atoms, which was consistent with the phenomenon classified by Robin and Day.^38^ As the potential becomes more anodic, reaching 1.0 V, the absorption bands of the cation radical gradually decrease, with a new broad band centered at around 770 nm grows. The disappearance of NIR absorption band can be attributed to the further oxidation from monocation radical species to the formation of dication in the 3,3′-bicarbazole segments.^35−37^ The UV–vis–NIR absorption changes in the **EC** film at various potentials were fully reversible, associated with strong color changes.

## Conclusions

In this work, we investigated the oxidation of carbazole-based polycyclic aromatic hydrocarbons **HPB-2Car** through traditional Scholl reactions and electrochemical oxidation, aided by cyclic voltammetry. Interestingly, our findings indicate that the oxidation reaction predominantly occurs at the carbazole functional group, driven by its rich electron density, which was further supported by theoretical studies. In addition, we conducted optical property, spectroelectrochemical property, and thermogravimetric analysis of the resulting dimeric to tetrameric products, demonstrating their potential for development as oligomer materials.

## Experimental Section

### Materials

All starting chemicals were purchased and used without further purification.

### 2,5-Diphenyl-3,4-bis(4-bromophenyl)cyclopentadienone (**TPCP-2Br**)

4,4′-Dibromobenzil (7.36 g, 20 mmol) and 1,3-diphenylacetone (4.21 g, 20 mmol) were mixed in ethanol (45 mL) and heated to 70 °C. Then, potassium hydroxide (1.33 g, 23.7 mmol) was dissolved in ethanol (15 mL), added dropwise to the reaction mixture, and heated at 80 °C for 3 h under a N_2_ atmosphere. The solution was cooled to room temperature and was then kept at 0 °C for 1 h. The solid was filtered off and washed with cold ethanol to afford a purple solid (9.60 g, 88%). ^1^H NMR (400 MHz, CDCl_3_, δ, ppm): 7.34 (d, *J* = 9.2 Hz, 4H, H_e_), 7.18–7.28 (m, 10H, H_b_ + H_c_ + H_a_), 6.79 (d, *J* = 8.8 Hz, 4H, H_d_). ^13^C NMR (100 MHz, CDCl_3_, ppm): 199.56 (C^1^), 152.56 (C^2^), 131.70 (C^3^), 131.51 (C^5^), 130.93 (C^4^), 130.22 (C^7^), 130.08 (C^9^), 128.22 (C^10^), 127.84 (C^6^), 125.94 (C^8^), 123.13 (C^11^). HRMS (FAB), Calcd for C_29_H_18_Br_2_O(M)^+^: 539.9724; Found: 541.9710. Anal. Calcd (%) for C_29_H_18_Br_2_O: C, 64.23%; H, 3.35%. Found: C, 63.97%; H, 3.42%.

### 2,5-Diphenyl-3,4-bis[4-(*N*-carbazolyl)phenyl]cyclopentadienone (**TPCP-2Car**)

**TPCP-2Br** (2.72 g, 5.0 mmol), 9*H*-carbazole (1.87 g, 11.2 mmol), palladium(II) acetate (0.45 g, 2.0 mmol), and sodium *tert*-butoxide (1.24 g, 12.9 mmol) were mixed in dry toluene (30 mL), and then tri-*tert*-butylphosphine (2 mL, 4.0 mmol) was added dropwise. The mixture was heated at 115 °C for 3 h under a N_2_ atmosphere. The solution was cooled and extracted with dichloromethane and deionized water. The organic layer was dried over anhydrous sodium sulfate, and the solvent was removed by rotary evaporation to get a crude product, which was reprecipitated by dichloromethane/hexane to afford a dark brown solid (3.18 g, 89%). ^1^H NMR (400 MHz, CDCl_3_, δ, ppm): 8.14 (d, *J* = 7.6 Hz, 4H, H_i_), 7.51 (d, *J* = 8.4 Hz, 4H, H_a_), 7.27–7.41 (m, 26H, H_b_ + H_c_ + H_d_ + H_e_ + H_f_ + H_g_ + H_h_). ^13^C NMR (100 MHz, CDCl_3_, δ, ppm): 199.93 (C^1^), 153.27 (C^7^), 140.42 (C^12^), 138.04 (C^11^), 132.05 (C^2^), 131.03 (C^10^), 130.52 (C^8^), 130.25 (C^4^), 128.31 (C^5^), 127.94 (C^6^), 126.44 (C^9^), 126.17 (C^14^), 125.92 (C^3^), 123.65 (C^17^), 120.46 (C^16^), 120.35 (C^15^), 109.57 (C^13^). HRMS (MALDI), Calcd for C_53_H_34_N_2_O(M + H)^+^: 715.2744; Found: 715.2761. Anal. Calcd (%) for C_53_H_34_N_2_O: C, 89.05%; H, 4.79%; N, 3.92%. Found: C, 88.51%; H, 4.83%; N, 3.77%.

### 1,2-Bis[4-(*N*-carbazolyl)phenyl]-3,4,5,6-tetraphenylbenzene (**HPB-2Car**)

**TPCP-2Car** (2.0 g, 2.8 mmol) and diphenylacetylene (0.54 g, 3.0 mmol) were dissolved in 4 mL of diphenyl ether. The mixture was heated at 260 °C for 5 days under a N_2_ atmosphere. The solution was cooled to r.t., 30 mL of methanol was added, and the resulting solid was separated by filtration. The crude product was purified using column chromatography with dichloromethane/hexane (3:7, v/v) to afford a white solid (1.68 g, 70%). ^1^H NMR (400 MHz, CDCl_3_, ppm): 8.07–8.10 (m, 4H, H_f_), 7.20–7.22 (m, 8H, H_c,d_), 7.15 (s, 8H, H_a_ + H_b_), 7.06–7.09 (m, 4H, H_e_), 6.96–7.01 (m, 10H, H_j_ + H_k_ + H_l_), 6.90–6.93 (m, 10H, H_g_ + H_h_ + H_i_). ^13^C NMR (100 MHz, CDCl_3_, ppm): 141.00 (C^14^), 140.74 (C^13^), 140.47 (C^5^), 140.43 (C^10^), 139.75 (C^4^), 134.96 (C^6^), 133.05 (C^11^), 131.67 (C^8^), 131.56 (C^7^), 127.07 (C^2^), 126.91 (C^3^), 126.06 (C^16^), 125.89 (C^12^), 125.72 (C^9^), 125.61 (C^1^), 123.31 (C^19^), 120.32 (C^18^), 119.84 (C^17^), 109.62 (C^15^). MS (MALDI): Calcd. For C_66_H_44_N_2_: 864.3521; Found: 864.3521. Anal. Calcd (%) for C_66_H_44_N_2_: C, 91.63%; H, 5.13%; N, 3.24%. Found: C, 91.83%; H, 5.11%; N, 3.16%.

### Oxidation C–C Coupling Attempts

#### Entry 1

A mixture of MoCl_5_ (0.36 g, 1.32 mmol) and **HPB-2Car** (0.09 g, 0.10 mmol) in dichloromethane (10 mL) was stirred overnight at room temperature under a N_2_ atmosphere. Then, methanol was added to quench, and the precipitate was washed by dichloromethane, methanol, and H_2_O repeatedly. Last, the precipitate was collected and dried to afford brown solid **M1**.

#### Entry 2

A mixture of aluminum chloride (0.46 g, 3.4 mmol) and cupric chloride (0.46 g, 3.4 mmol) in carbon disulfide (30 mL) was stirred for 20 min at room temperature under a N_2_ atmosphere, and then, **HPB-2Car** (0.11 g, 0.13 mmol) was added into the mixture. The reaction mixture was stirred for 5 days at room temperature. The reaction solution was quenched with methanol, and the precipitate was filtered off and repeatedly washed with methanol. The precipitate was collected and dried to afford reddish brown solid **M2**.

#### Entry 3

Compound **HPB-2Car** (0.10 g, 0.1 mmol) was dissolved in dry dichloromethane (30 mL). The solution was degassed via bubbling nitrogen for 30 min. Then, ferric chloride (0.64 g, 3.87 mmol) in nitromethane (5 mL) was added slowly via a syringe. The reaction mixture was stirred at room temperature for 13 days under a N_2_ atmosphere. The reaction solution was quenched with methanol, and the precipitate was filtered off and repeatedly washed with methanol. The yellow gray precipitate was collected and dried to afford earthy gray solid **M3**.

#### Entry 4

Compound **HPB-2Car** (0.10 g, 0.1 mmol) was dissolved in 1,2-dichloroethane (30 mL). The solution was heated to 80 °C and degassed via bubbling nitrogen for 30 min. Then, ferric chloride (0.62 g, 3.74 mmol) in nitromethane (6 mL) was added slowly via a syringe. The reaction mixture was heated at 85 °C for 7 days under a N_2_ atmosphere. The reaction solution was quenched with methanol, and the precipitate was filtered off and repeatedly washed with methanol. The precipitate was collected and dried to afford black solid **M4**. MS (MALDI): calcd. For C_132_H_86_N_4_, C_198_H_128_N_6_, C_264_H_170_N_8_: 1726.69, 2589.02, 3451.35; found:1725.7, 2586.9, 3448.2.

#### Entry 5

A mixture of aluminum chloride (0.46 g, 3.4 mmol) and copper(II) trifluoromethanesulfonate (1.25 g, 3.4 mmol) in carbon disulfide (25 mL) was stirred for 45 min at room temperature under a N_2_ atmosphere, and then, **HPB-2Car** (0.10 g, 0.1 mmol) was added into the mixture. The reaction mixture was stirred for 13 days at room temperature. The reaction solution was quenched with methanol, and the precipitate was filtered off and repeatedly washed with methanol. The precipitate was collected and dried to afford the ochre solid **M5**.

#### Entry 6

Compound **HPB-2Car** (0.11 g, 0.13 mmol) and 2,3-dichloro-5,6-dicyano-*p*-benzoquinone (0.17 g, 0.75 mmol) were added to dry dichloromethane (40 mL). The solution was degassed via bubbling nitrogen for 30 min. Then, trifluoromethanesulfonic acid (0.13 mL, 1.4 mmol) was added slowly via a syringe in an ice bath. The reaction mixture was stirred at room temperature for 12 days under a N_2_ atmosphere. The reaction solution was quenched with methanol, and the precipitate was filtered off and repeatedly washed with methanol. The dark brown precipitate was collected and dried to afford **M6** (51.4 mg). MS (MALDI): calcd. For C_132_H_86_N_4_: 1726.69; found:1725.7.

#### Entry 7

Compound **HPB-2Car** (0.11 g, 0.12 mmol) was dissolved in 1,2-dichloroethane (10 mL). Then, PhI(O_2_CCF_3_)_2_ (0.74 g, 1.71 mmol) and BF_3_·Et_2_O (0.2 mL, 4.01 mmol) were added slowly. The reaction mixture was stirred at −40 °C for 3 h under a N_2_ atmosphere. The reaction solution was quenched with methanol, and the precipitate was filtered off and repeatedly washed with methanol. The precipitate was collected and dried to afford a dark brown solid **M7**.

#### Entry 8

The dimer of **HPB-2Car** was electrochemically synthesized from **HPB-2Car** (10^–4^ M in electrolyte solution) in a three-electrode system using ITO-coated glass as the working electrode, a platinum wire as the counter electrode, and a Ag/AgCl reference electrode. Dichloromethane with supporting electrolyte (0.1 M TBAP) was used as electrolyte solution. Electrochemical oxidation was conducted by using multicycle CV in the potential range from 0 to 1.4 V. Then use the amperometric method to provide a voltage of 1.6 V for 60 min to obtain a film **EC** on the ITO. MS (MALDI): calcd. For C_132_H_86_N_4_: 1726.69; found:1725.7.

### Instrumentations

^1^H and ^13^C nuclear magnetic resonance (NMR) spectra were measured on a Bruker AVANCE-400 FT-NMR, operating at frequencies of 400 MHz for ^1^H and 100 MHz for ^13^C measurements with CDCl_3_ as the solvent. All measurements were carried out at standard conditions at room temperature. Chemical shifts are reported in parts per million (ppm, δ) relative to the solvent residual proton (CDCl_3_, δ7.26) and carbon (CDCl_3_, δ77.2) signals. Peak multiplicity was reported as follows: s, singlet; d, doublet; t, triplet; m, multiplet. Molecular weights were obtained using a matrix-assisted laser desorption/ionization time-of-flight instrument (MALDI-TOF; Bruker, New ultrafleXtremeTM, Bremen, D.E.), and FAB mass spectra were obtained on a JMS-700 double focusing magnetic sector mass spectrometer (JEOL, Tokyo, Japan). Fourier transform infrared (FTIR) spectra were measured on a Spectrum 100 FT-IR Spectrometer, PerkinElmer Inc. Raman spectra were obtained using a HORIBA iHR550 Raman spectrometer at an excitation source of 532 nm with the intensity ranging from 6.25–25 mW and 120 s acquisition time. The scanning electron microscope (SEM) images were obtained using a field-emission scanning electron microscope (FESEM), Ultra Plus-Carl Zeiss. Work functions were measured on an AC-2 Photoemission Yield Spectroscopy in the Air (PYSA), RIKEN KEIKI CO., Ltd. UV–vis absorption spectra were recorded on an Agilent Technologies Model Cary 8454 UV–visible spectroscopy system. Photoluminescence (PL) spectra were recorded on a HITACHI F-4500 FL spectrophotometer. Cyclic voltammetry (CV) was performed on a CH Instruments Model CHI660E electrochemical workstation, and measurements were carried out in dichloromethane containing 0.1 M TBAP as the supporting electrolyte (scan rate: 50 mV s^–1^). An ITO electrode was used as a working electrode, a platinum wire as a counter electrode, and Ag/AgCl as a reference electrode. Thermogravimetric analysis (TGA) measurements were performed on a PerkinElmer Pyris 1 TGA instrument. Experiments were carried out on approximately 5 mg samples heated in flowing nitrogen (flow rate = 20 cm^3^/min) at a heating rate of 10 °C/min.

## Supporting Information Available

Characterization data: ^1^H NMR spectra, ^13^C NMR spectra, DEPT-135 spectra, FAB MS spectra, MALDI-TOF MS spectra, FESEM images, relative Gibbs free energies, PSE scan, FTIR spectra, Raman spectra, TGA thermograms, work function, additional DFT results, and calculated electronic energies, zero-point energy corrected energies, electronic and thermal enthalpies, Gibbs free energies of cyclization pathways 1 and 2 and dimerization (PDF)
