# Supplementary material for: Electroactive Carbazole-Based Polycyclic Aromatic Hydrocarbons: Synthesis, Photophysical Properties, and Computational Studies
Source: ACS Omega. 2024 Jun 18;9(27):29379–90. doi: 10.1021/acsomega.4c01434 (PMC11238223; doi:10.1021/acsomega.4c01434)
Supplement: Supplementary file 1 — ao4c01434_si_001.pdf [file ao4c01434_si_001.pdf]

Supporting Information

Electroactive Carbazole-Based Polycyclic Aromatic Hydrocarbons: Synthesis, Photophysical Properties, and Computational Studies

Hui Qi Wong[1,2,3,†], Ting-Hsuan Lin[1,4,†], Jian-Ming Liao[5,†], Septia Kholimatussadiah[6], Febri Baskoro[1], Hui-Hsu Gavin Tsai[5,7,] and Hung-Ju Yen[1,*]*

[1] Institute of Chemistry, Academia Sinica, 128 Academia Road, Section 2, Nankang, Taipei 11529, Taiwan

[2] Sustainable Chemical Science and Technology Program, Taiwan International Graduate Program (TIGP), Academia Sinica and National Taiwan University, Taipei 11529, Taiwan

[3] Department of Chemical Engineering, National Taiwan University, Taipei 10617, Taiwan

[4] Department of Chemical Engineering, National Taiwan University of Science and Technology, Taipei 10607, Taiwan

[5] Department of Chemistry, National Central University, No. 300, Zhongda Rd., Zhongli District, Taoyuan City, 32001, Taiwan

[6] Nano Science and Technology Program, TIGP, Academia Sinica and National Taiwan University, Taipei 11529, Taiwan

[7] Research Center of New Generation Light Driven Photovoltaic Module, National Central University, No. 300, Zhongda Road, Zhongli District, Taoyuan City, 32001, Taiwan

Corresponding authors: hhtsai@cc.ncu.edu.tw; hjyen@gate.sinica.edu.tw

[†] Equal contribution

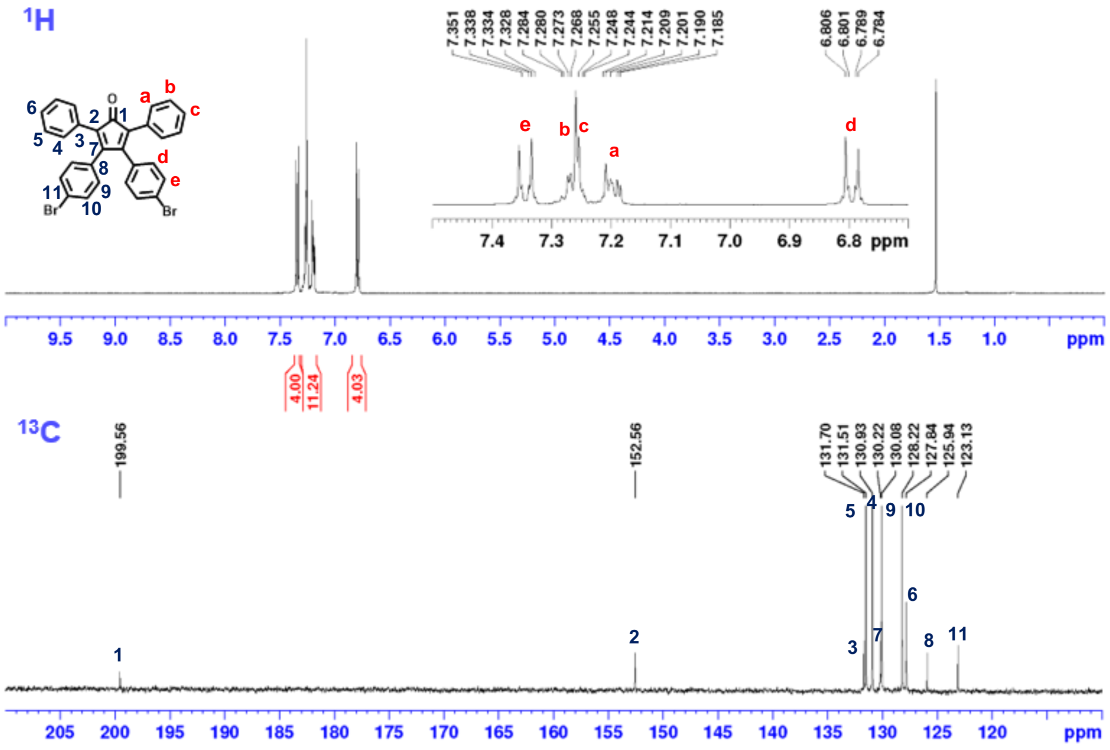

Figure S1. ^{1}H and ^{13}C NMR spectra of **TPCP-2Br** in $CDCl_3$.

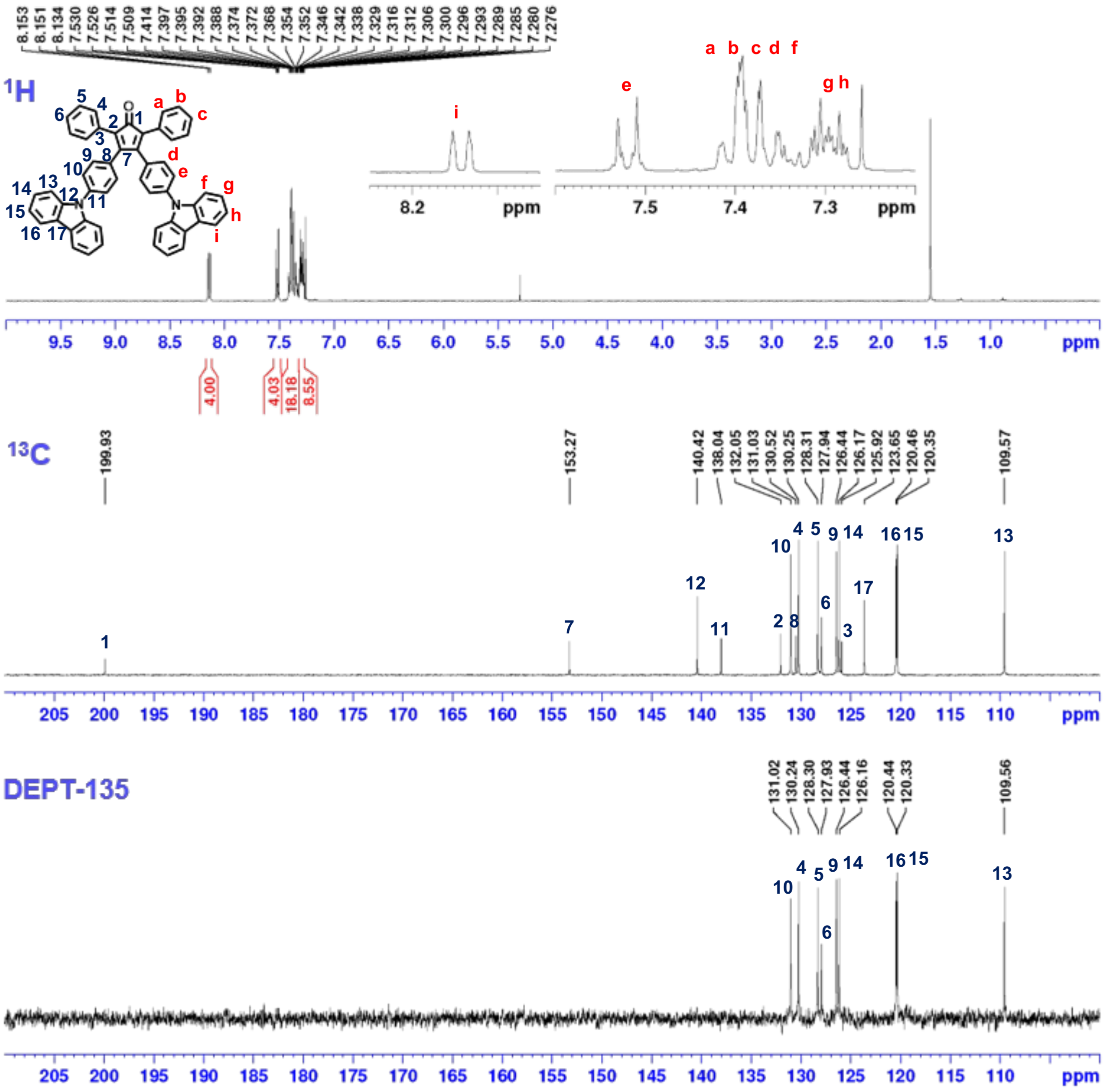

Figure S2. ¹H, ¹³C NMR, and DEPT-135 spectra of **TPCP-2Car** in $CDCl_3$.

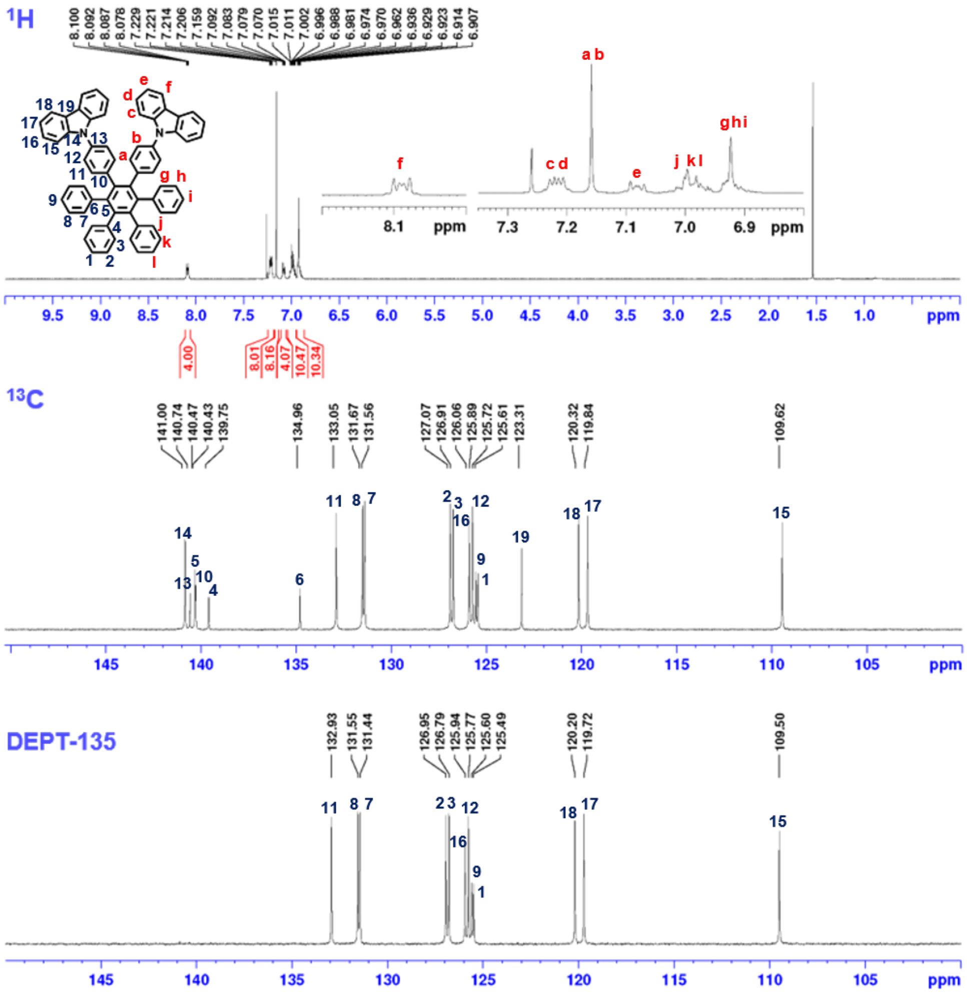

Figure S3. ^{1}H, ^{13}C NMR, and DEPT-135 spectra of **HPB-2Car** in $CDCl_3$.

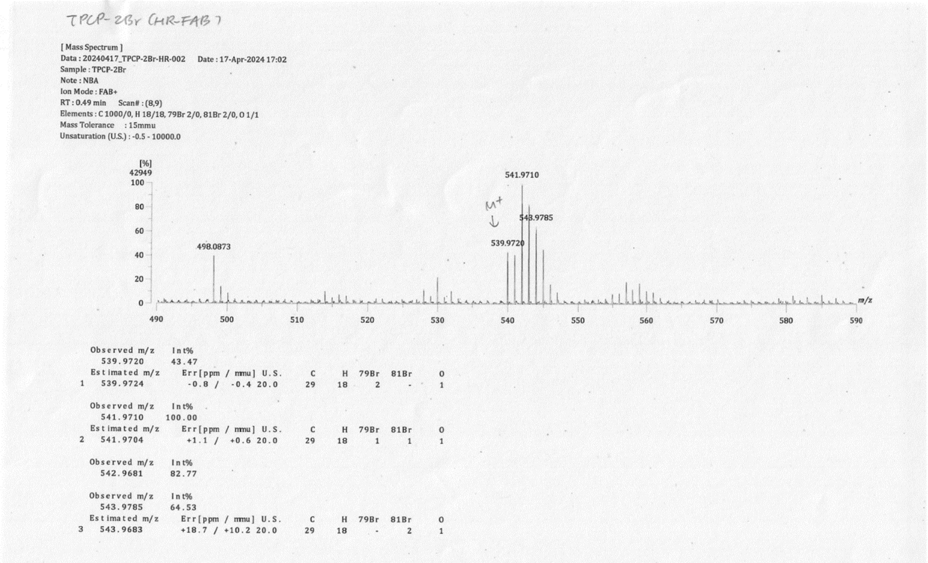

Figure S4. FAB MS spectrum of **TPCP-2Br**.

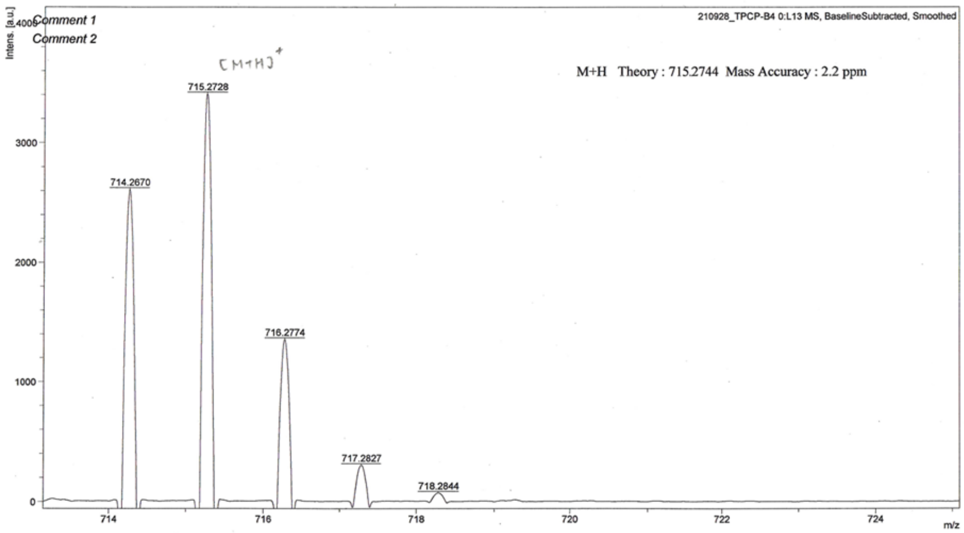

Figure S5. MALDI-TOF MS spectrum of **TPCP-2Car**.

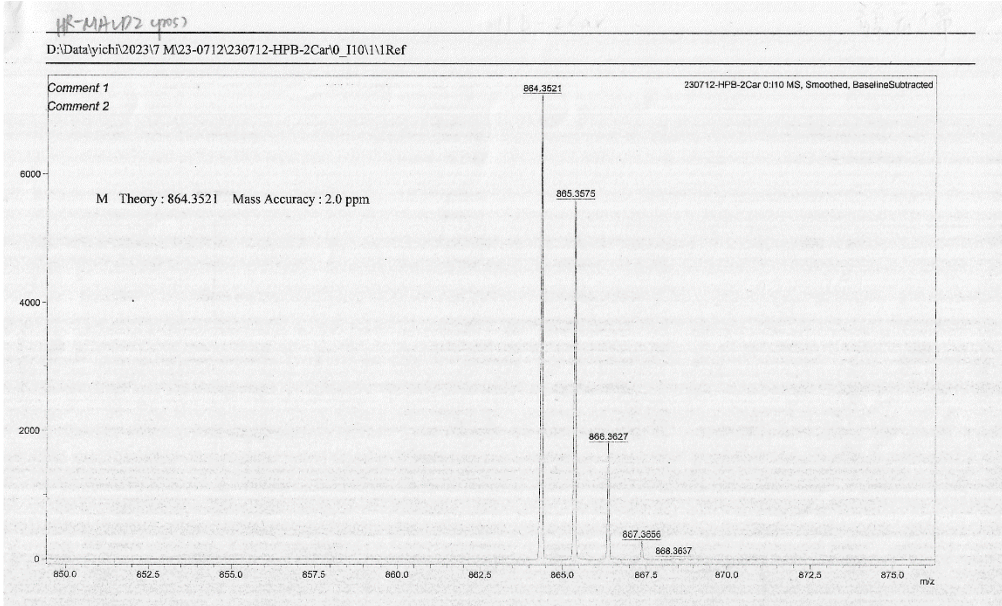

Figure S6. MALDI-TOF MS spectrum of **HPB-2Car**.

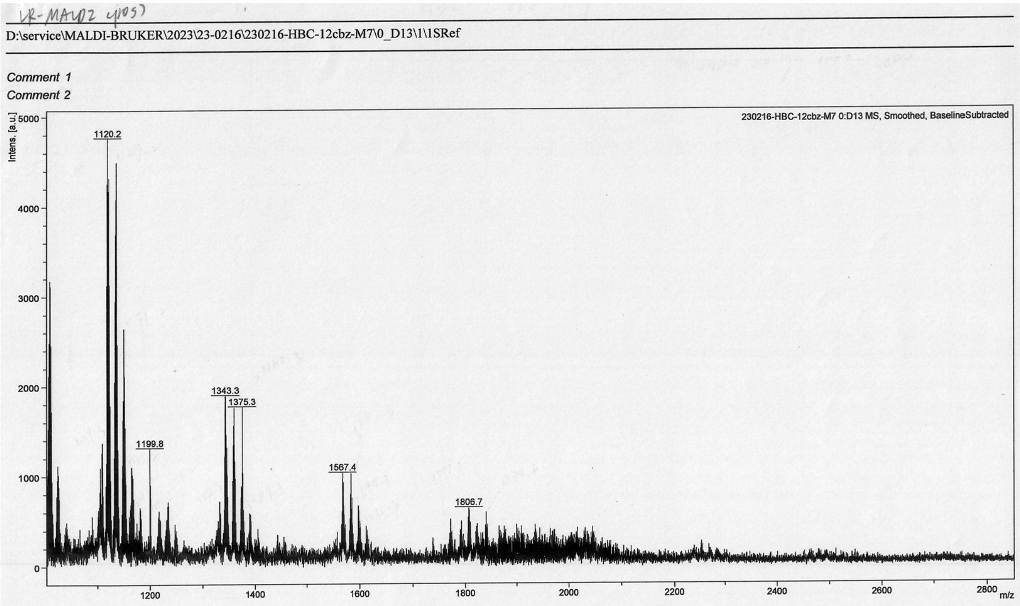

Figure S7. MALDI-TOF MS spectrum of **M1**.

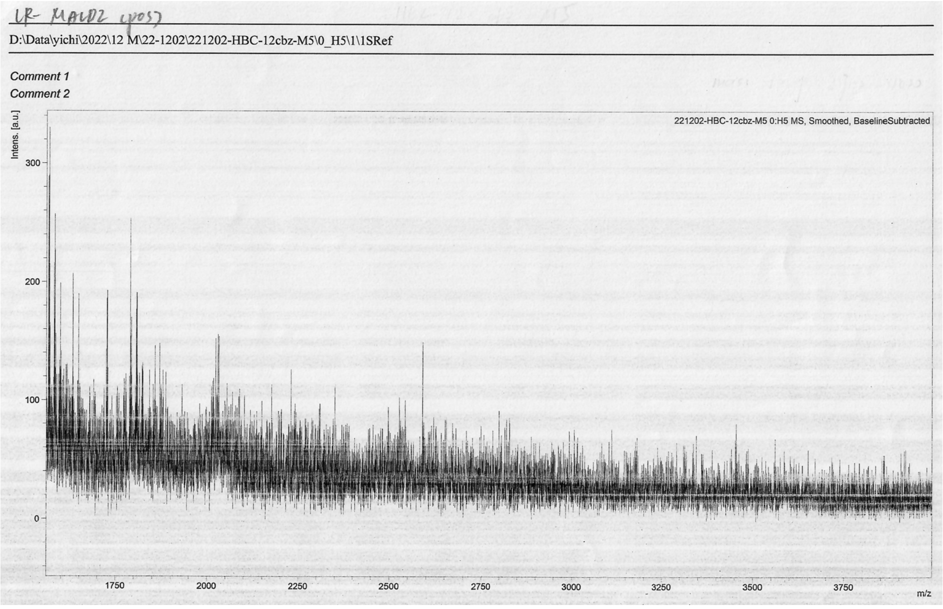

Figure S8. MALDI-TOF MS spectrum of **M2**.

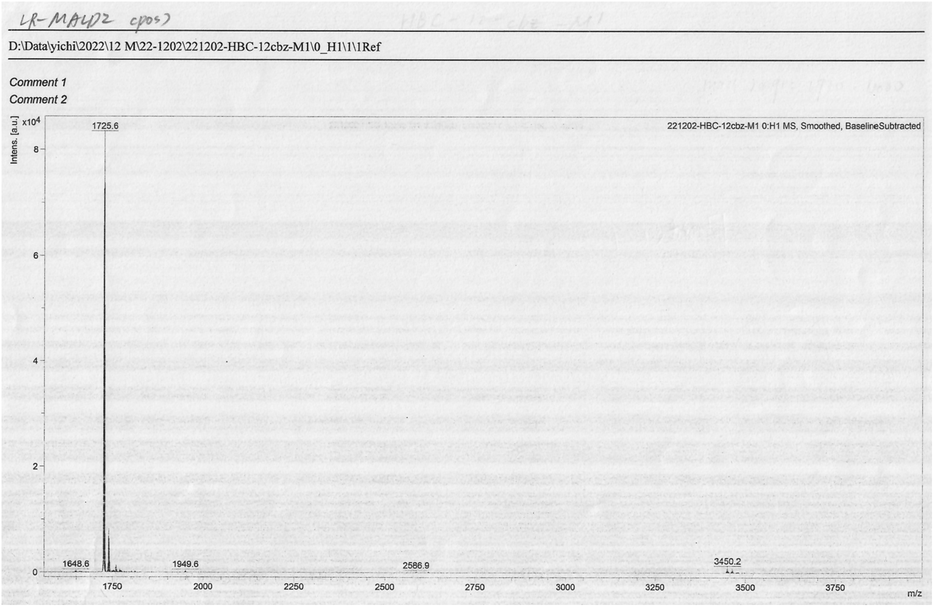

Figure S9. MALDI-TOF MS spectrum of **M3**.

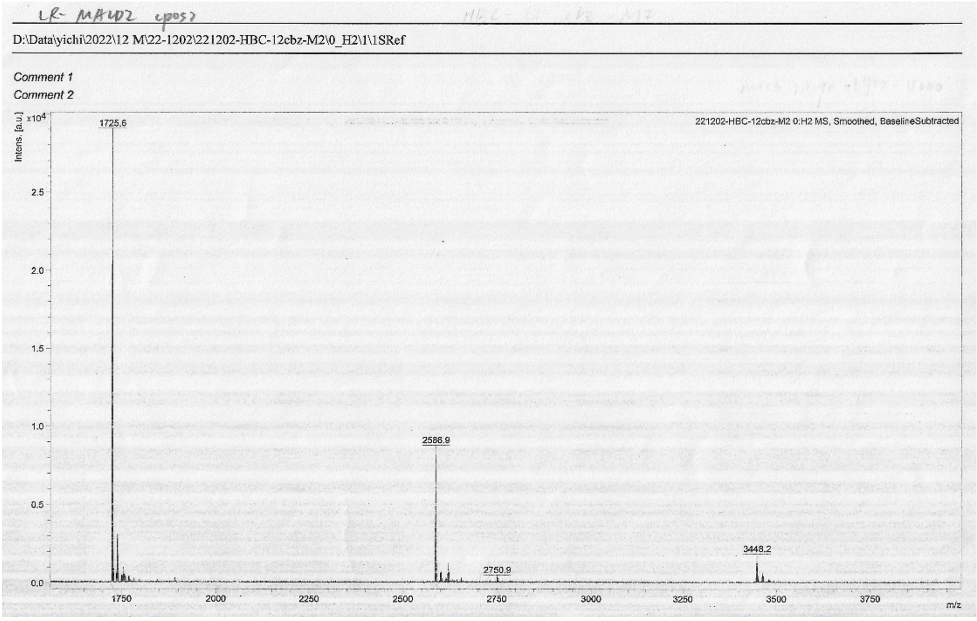

Figure S10. MALDI-TOF MS spectrum of **M4**.

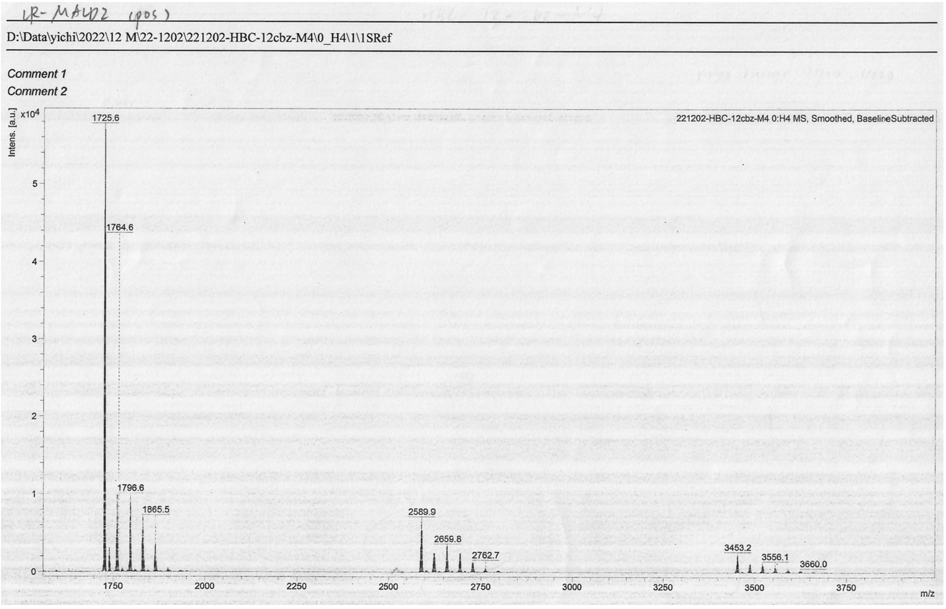

Figure S11. MALDI-TOF MS spectrum of **M5**.

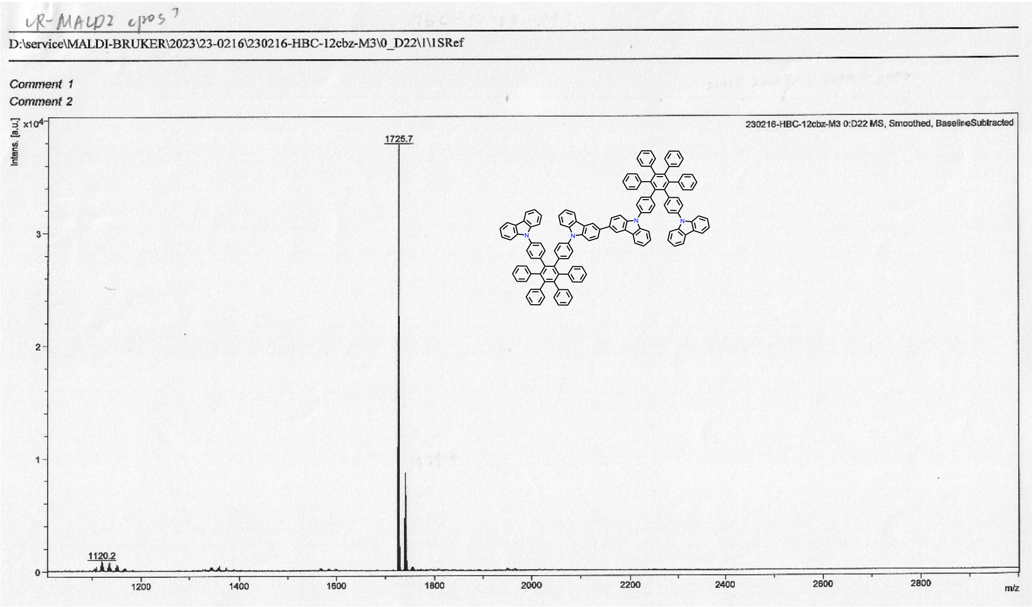

Figure S12. MALDI-TOF MS spectrum of **M6**.

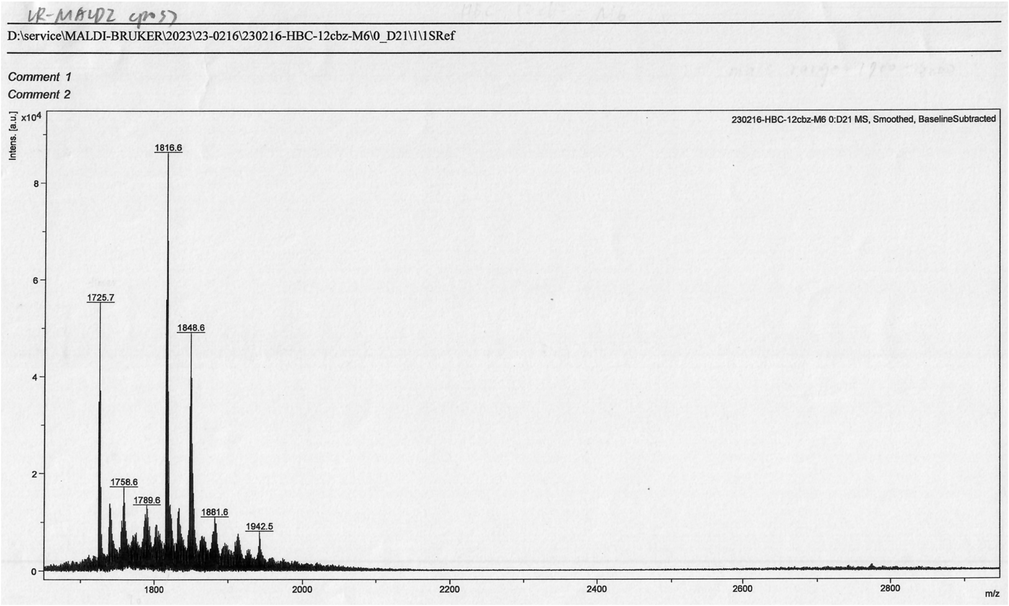

Figure S13. MALDI-TOF MS spectrum of **M7**.

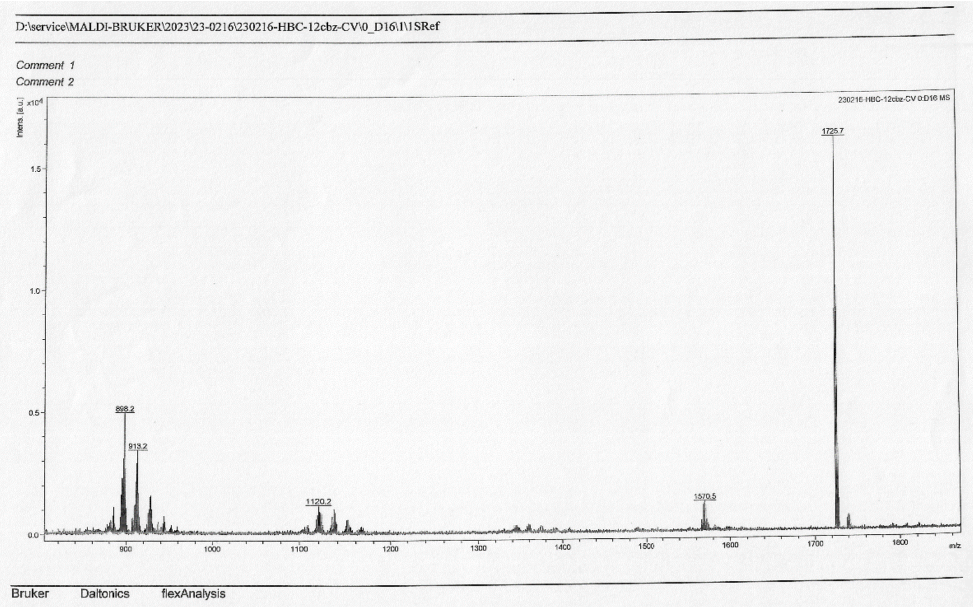

Figure S14. MALDI-TOF MS spectrum of **EC**.

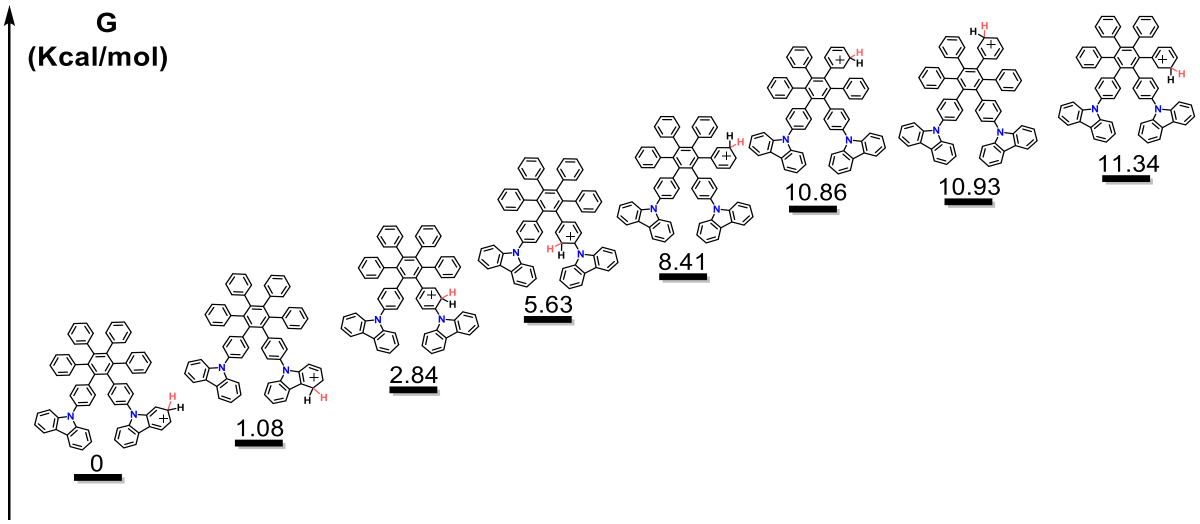

Figure S15. The relative Gibbs free energies (kcal/mol, 298 K) of eight isomers of protonated **HPB-2Car**.

Table S1. Cyclization Pathway 1 and Dimerization: Calculated electronic energies (E), zero-point energy corrected energies (E + ZPE), electronic and thermal enthalpies (H), Gibbs free energies (G) of calculated at B3LYP-D3/6-31G (d,p) level with C-PCM solvent model of dichloromethane.

Molecule	E (Hartree)	E + ZPE (Hartree)	H (Hartree)	G (Hartree)
**Cyclization Pathway 1**				
$^{CYC1}IM1_{AC}$	-2651.91706	-2651.004948	-2650.952481	-2651.093135
$^{CYC1}TS_{AC}$	-2651.87153	-2650.959849	-2650.90813	-2651.047553
$^{CYC1}IM2_{AC}$	-2651.873613	-2650.960749	-2650.908688	-2651.050588
$^{CYC1}IM1_{RC}$	-2651.325276	-2650.425200	-2650.372927	-2650.514331
$^{CYC1}TS_{RC}$	-2651.246303	-2650.347559	-2650.296397	-2650.436241
$^{CYC1}IM2_{RC}$	-2651.247613	-2650.348108	-2650.296232	-2650.437951
^{CYC1}P	-2650.316039	-2649.436843	-2649.385866	-2649.524535
**Dimerization**				
$^{DIM}IM1_{AC}$	-2651.916287	-2651.005813	-2650.952732	-2651.097655
$^{DIM}TS_{AC}$	-5303.410835	-5301.599308	-5301.493585	-5301.762361
$^{DIM}IM2_{AC}$	-5303.414191	-5301.601207	-5301.495387	-5301.763249
$^{DIM}IM1_{RC}$	-2651.325276	-2650.4252	-2650.372927	-2650.514331
$^{DIM}TS_{RC}$	-5302.618778	-5300.817152	-5300.71174	-5300.979567
$^{DIM}IM2_{RC}$	-5302.631419	-5300.827616	-5300.722132	-5300.989809
^{DIM1}P	-5301.840909	-5300.061943	-5299.956866	-5300.223444
**HPB-2Car**	-2651.514436	-2650.614825	-2650.562256	-2650.706423
CF_3SO_3H	-962.061839	-962.023936	-962.015129	-962.057134
$CF_3SO_3^-$	-961.641819	-961.615452	-961.607248	-961.647998
DDQ	-1485.144742	-1485.08237	-1485.069047	-1485.122648
$DDQH^{\cdot}$	-1485.76422	-1485.690484	-1485.677113	-1485.730787
$DDQH_2$	-1486.40549	-1486.319445	-1486.305656	-1486.359197

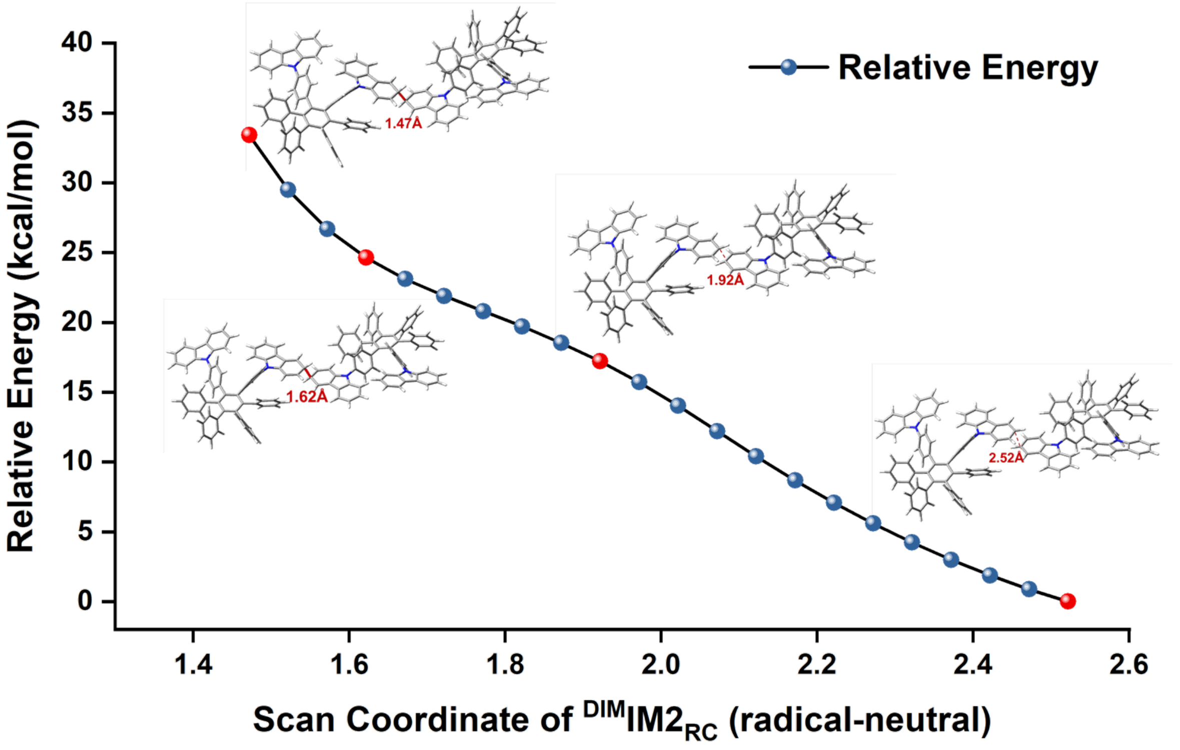

Figure S16. Potential Energy Surface (PSE) Scan for ^{DIM}**IM2**$_{RC}$ (radical-neutral). The X-axis of the graph corresponds to the C-C bond length in Ångstroms (Å). The scan conducted focused on the C-C bond distances ranging from 2.50 Å to 1.40 Å, in a step of 0.05 Å.

Table S2. Cyclization Pathway 2: Electronic energies (E), zero-point energy corrected energies (E + ZPE), electronic and thermal enthalpies (H), Gibbs free energies (G) of calculated at B3LYP-D3/6-31G (d,p) level with C-PCM solvent model DCM.

Molecule	E (Hartree)	E + ZPE (Hartree)	H (Hartree)	G (Hartree)
**Cyclization Pathway 2**				
$^{CYC2}IM1_{AC}$	-2651.91706	-2651.004948	-2650.952481	-2651.093135
$^{CYC2}TS_{AC}$	-2651.868359	-2650.957103	-2650.905211	-2651.046149
$^{CYC2}IM2_{AC}$	-2651.869167	-2650.957011	-2650.904775	-2651.046944
$^{CYC2}IM1_{RC}$	-2651.325276	-2650.425200	-2650.372927	-2650.514331
$^{CYC2}TS_{RC}$	-2651.243297	-2650.345274	-2650.293816	-2650.434488
$^{CYC2}IM2_{RC}$	-2651.244582	-2650.345479	-2650.293468	-2650.435705
^{CYC2}P	-2650.313175	-2649.434585	-2649.383293	-2649.524303

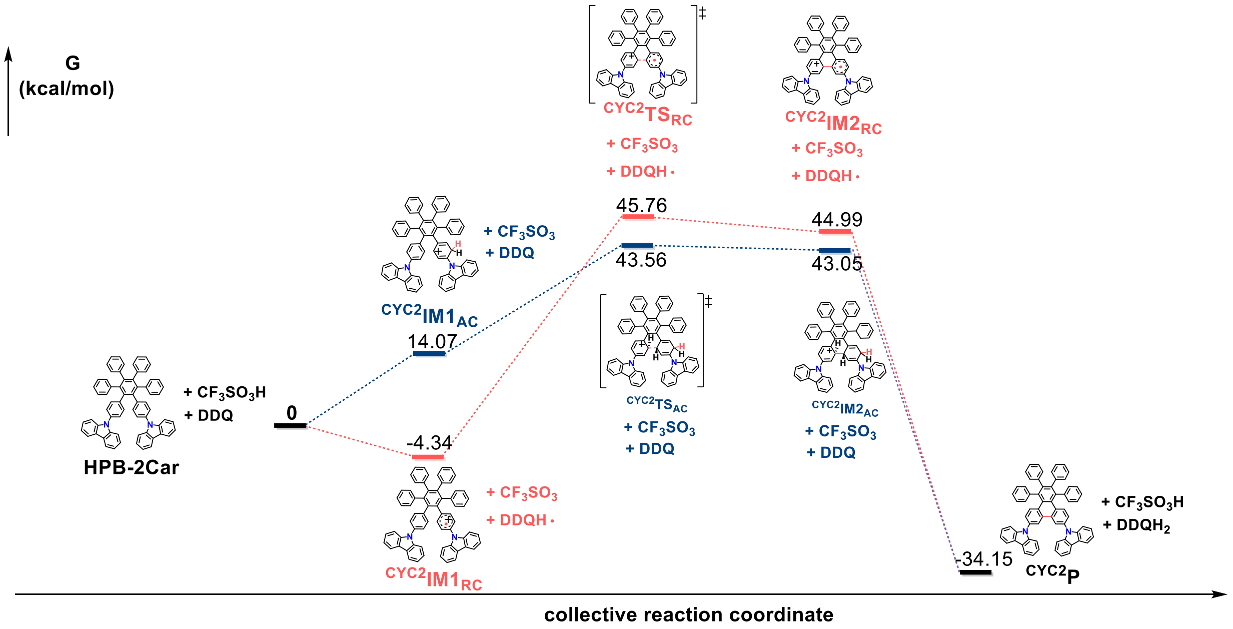

Figure S17. DFT calculated relative Gibbs free-energies. (ΔG at 298 K) for the cyclization pathway 2 of **HPB-2Car** involving the arenium cation (blue bars) and radical cation (green bars) mechanisms.

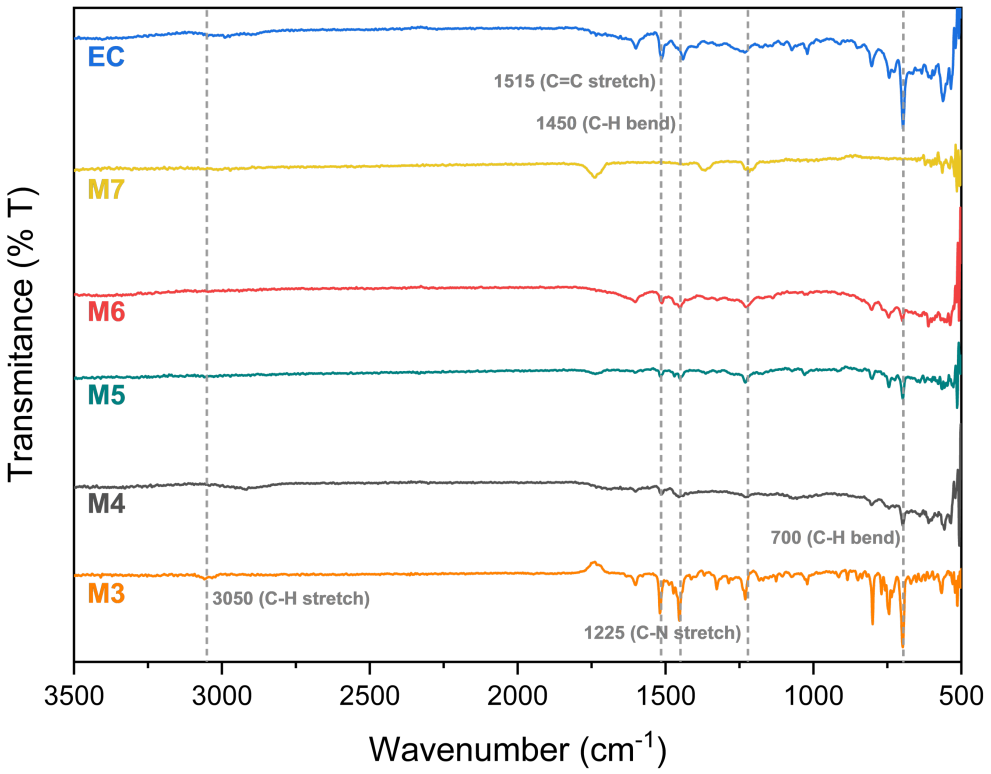

Figure S18. FTIR spectra of **M3**-**M7** and **EC**.

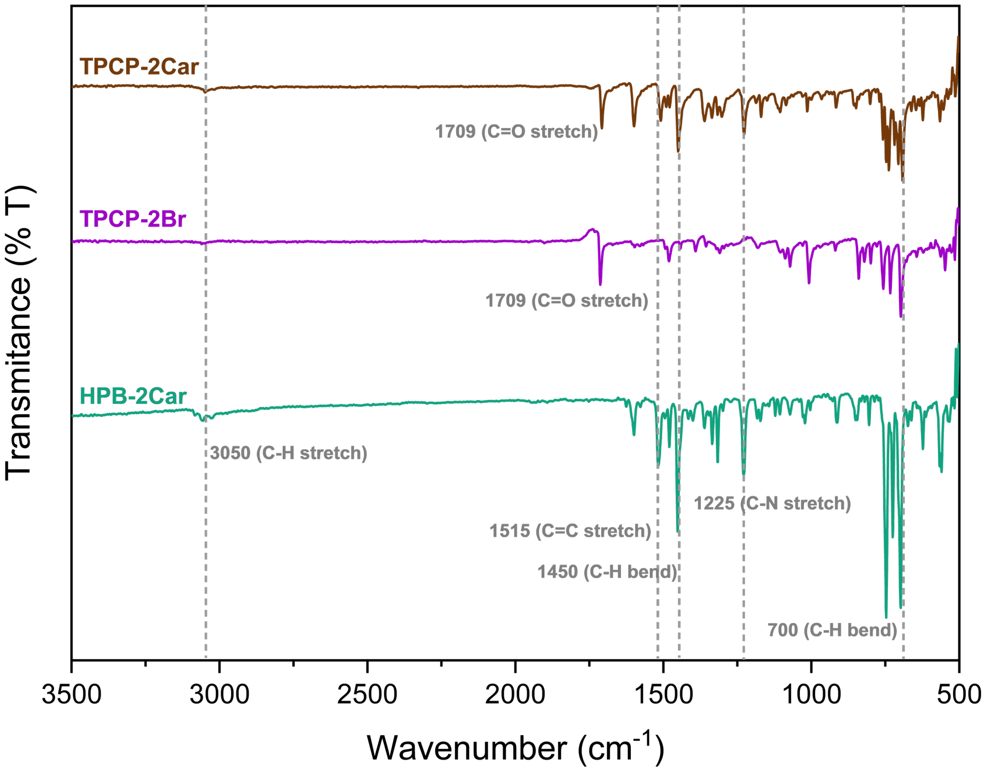

Figure S19. FTIR spectra of **TPCP-2Car**, **TPCP-2Br** and **HPB-2Car**.

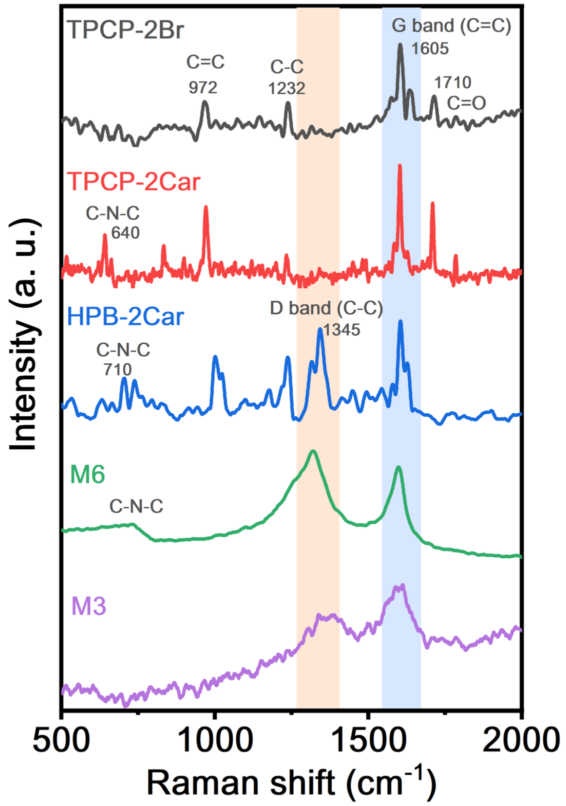

Figure S20. Raman spectra of **TPCP-2Br**, **TPCP-2Car**, **HPB-2Car**, **M3**, and **M6**

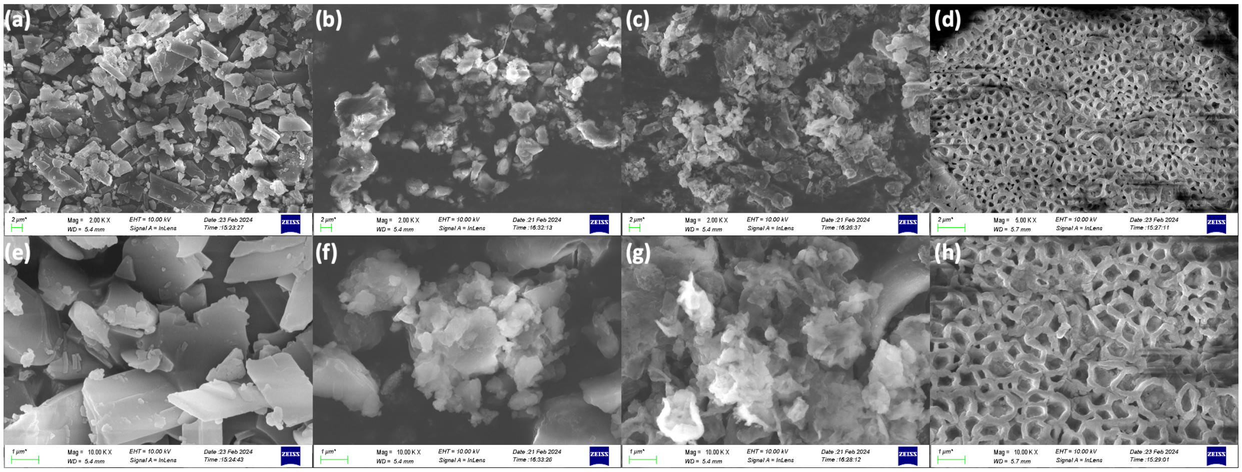

Figure S21. The FESEM images of (a & e) **HPB-2Car**; (b & f) **M4**; (c & g) **M6**; (d & h) **EC** at different magnification, respectively.

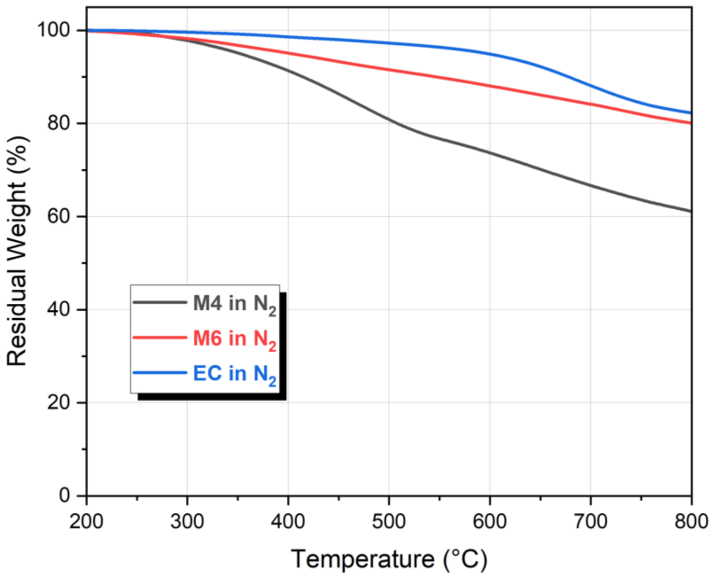

Figure S22. TGA thermograms of **M4**, **M6** and **EC**.

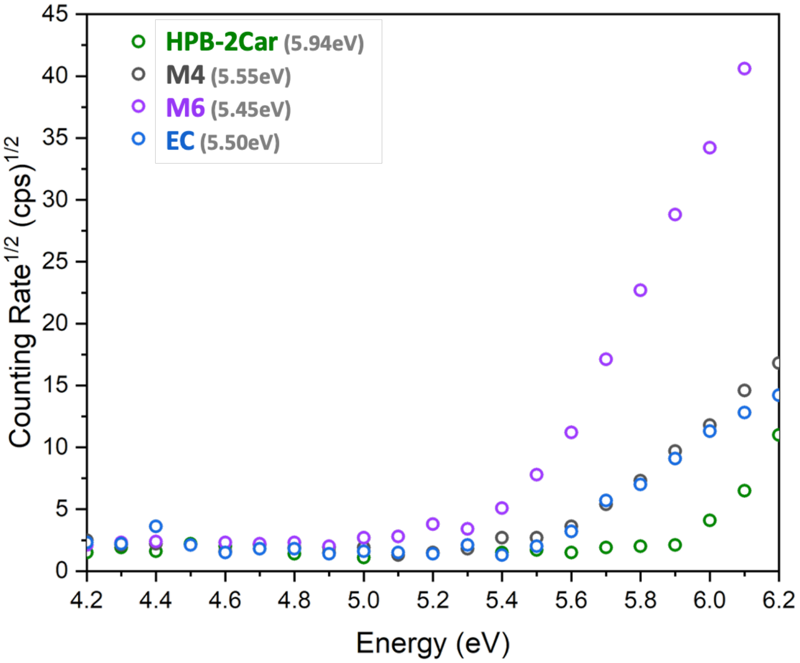

Figure S23. Work function of **HPB-2Car** and it dimerized products.

Supporting Reference

1. Meitinger, N.; Mengele, A. K.; Witas, K.; Kupfer, S.; Rau, S.; Nauroozi, D. Tetraaryl Cyclopentadienones: Experimental and Theoretical Insights into Negative Solvatochromism and Electrochemistry. *Eur. J. Org. Chem.* **2020,** *2020* (42), 6555-6562.
2. Hill, L. L.; Moore, L. R.; Huang, R.; Craciun, R.; Vincent, A. J.; Dixon, D. A.; Chou, J.; Woltermann, C. J.; Shaughnessy, K. H. Bulky Alkylphosphines with Neopentyl Substituents as Ligands in the Amination of Aryl Bromides and Chlorides. *J. Org. Chem.* **2006,** *71* (14), 5117-5125.
3. Maly, K. E.; Gagnon, E.; Maris, T.; Wuest, J. D. Engineering Hydrogen-Bonded Molecular Crystals Built from Derivatives of Hexaphenylbenzene and Related Compounds. *J. Am. Chem. Soc.* **2007,** *129* (14), 4306-4322.
